# Intraparenchymal convection enhanced delivery of AAV in sheep to treat Mucopolysaccharidosis IIIC

**DOI:** 10.1186/s12967-023-04208-1

**Published:** 2023-07-05

**Authors:** Claire O’Leary, Gabriella Forte, Nadia L. Mitchell, Amir Saam Youshani, Adam Dyer, Martin P. Wellby, Katharina N. Russell, Samantha J. Murray, Nelly Jolinon, Simon A Jones, Kevin Stacey, Daniel M. Davis, Els Henckaerts, David N. Palmer, Ian Kamaly-Asl, Brian W. Bigger

**Affiliations:** 1 Stem Cell & Neurotherapies, Division of Cell Matrix Biology and Regenerative Medicine, School of Biological Sciences, Faculty of Biology Medicine and Health, University of Manchester, Manchester, UK; 2 The Geoffrey Jefferson Brain Research Centre, University of Manchester, Manchester Academic Health Science Centre, Northern Care Alliance, Manchester, UK; 3 Department of Molecular Biosciences, Faculty of Agriculture and Life Sciences, Lincoln University, Lincoln, 7647 New Zealand; 4 Department of Radiology, University of Otago, Christchurch, 8140 New Zealand; 5 Department of Infectious Diseases, School of Immunology and Microbial Sciences, King’s College London, London, UK; 6 Manchester Centre for Genomic Medicine, Willink Unit, Manchester University NHS Foundation Trust, Manchester, UK; 7 Lydia Becker Institute of Immunology and Inflammation, Division of Infection, Immunity and Respiratory Medicine, School of Biological Sciences, Faculty of Biology Medicine and Health, Manchester Collaborative Centre for Inflammation Research, University of Manchester, Manchester, UK; 8 Department of Life Sciences, Imperial College London, Sir Alexander Fleming Building, South Kensington, London, UK; 9 Laboratory of Viral Cell Biology & Therapeutics, Department of Cellular and Molecular Medicine and Department of Microbiology, Immunology and Transplantation, KU Leuven, Leuven, Belgium; 10 Department of Paediatric Neurosurgery, Royal Manchester Children’s Hospital, Manchester, UK

**Keywords:** AAV gene therapy, Convection Enhanced Delivery, HGSNAT, Large animal, Mucopolysaccharidosis

## Abstract

**Background:**

Mucopolysaccharidosis IIIC (MPSIIIC) is one of four Sanfilippo diseases sharing clinical symptoms of severe cognitive decline and shortened lifespan. The missing enzyme, heparan sulfate acetyl-CoA: α-glucosaminide-N-acetyltransferase (HGSNAT), is bound to the lysosomal membrane, therefore cannot cross the blood-brain barrier or diffuse between cells. We previously demonstrated disease correction in MPSIIIC mice using an Adeno-Associated Vector (AAV) delivering HGSNAT via intraparenchymal brain injections using an AAV2 derived AAV-truetype (AAV-TT) serotype with improved distribution over AAV9.

**Methods:**

Here, intraparenchymal AAV was delivered in sheep using catheters or Hamilton syringes, placed using Brainlab cranial navigation for convection enhanced delivery, to reduce proximal vector expression and improve spread.

**Results:**

Hamilton syringes gave improved AAV-GFP distribution, despite lower vector doses and titres. AAV-TT-GFP displayed moderately better transduction compared to AAV9-GFP but both serotypes almost exclusively transduced neurons. Functional HGSNAT enzyme was detected in 24-37% of a 140g gyrencephalic sheep brain using AAV9-HGSNAT with three injections in one hemisphere.

**Conclusions:**

Despite variabilities in volume and titre, catheter design may be critical for efficient brain delivery. These data help inform a clinical trial for MPSIIIC.

**Supplementary Information:**

The online version contains supplementary material available at 10.1186/s12967-023-04208-1.

## Introduction

MPSIIIC is one of four Sanfilippo diseases (MPSIIIA-D), sharing clinical signs and symptoms, caused by mutations in the gene encoding for the enzyme heparan sulfate acetyl-CoA: α-glucosaminide N-acetyltransferase (HGSNAT). HGSNAT deficiency affects lysosomal catabolism of heparan sulfate (HS), resulting in widespread CNS pathology in infants and children, leading to behavioural problems, cognitive decline, dementia and death before adulthood [1]. There are no existing treatments for MPSIIIC and the development of treatments is difficult, as unlike MPS IIIA, B or D, the deficient enzyme in MPSIIIC cannot cross the blood brain barrier or diffuse between cells as it is bound to the lysosomal membrane [2]. Despite this, we have previously shown that an AAV based therapy for MPSIIIC can reduce storage of HS and correct working memory defects in an MPSIIIC murine model via bilateral intracranial injections into the brain parenchyma [3]. However, the architecture and volume of a small murine brain is considerably different from a human brain. This difference in brain structure from a lissencephalic mouse brain to a gyrencephalic human brain may be responsible for the limited efficacy in MPSIIIA and IIIB patients using intraparenchymal injection of AAV [4, 5], despite success in treating a mouse model with the same clinical vector [6]. Thus, large animal experiments are essential in optimising widespread distribution needed for clinical efficacy. Due to the inability of HGSNAT to cross-correct cells, delivery of vector into the brain needs to target the maximum number of cells.

Following the success of intravenous AAV9-mediated delivery of SMN1 for spinal muscular atrophy, many groups have adopted this approach for brain diseases, but even in MPSIIIA, which is notably a cross-correctable disease, only patients treated with the highest doses and at the youngest ages (<30 months) are responding to treatment [7].

Indeed, delivery of AAV9 via intravenous and CSF delivery routes in nonhuman primates and sheep, appears to mainly result in transduction of glial cells, with commensurate loss of expression over time [8]. In contrast AAV delivered via the intraparenchymal route typically transduces neurons, which should permit longevity of treatment effect due to low turnover of neurons in the brain.

In addition, the intraparenchymal delivery route also suffers from significant overexpression of AAV product proximal to the injection site, with distal distribution typically limited to a few millimetres from the site. This is an issue for diseases with global brain pathology such as lysosomal storage diseases, leukodystrophies or Alzheimer’s disease, where maximum distribution of vector is required. Direct intracerebral infusion of vector via convection enhanced delivery (CED) is optimal [9] allowing direct brain infusion of small and large macromolecules, with the option of real-time monitoring of flow rates and allowing for pressure-driven delivery to optimise interstitial delivery of drugs [10]. However, a safe and robust technique for CED is required, especially in light of the FDA halting the Voyager study (NCT01973543) in which VY-AADC01 was administered into the putamen by MRI-guided convective infusion in patients with Parkinson’s disease, and also the Lysogene study (NCT03612869), delivering AAVrh10 expressing SGSH under the CAG promoter in patients with MPSIIIA, following MRI findings at the intracerebral injection sites.

CED uses a hydrostatic pressure gradient to maximise spread of compounds throughout the brain and, crucially, high volumes can be delivered. It also prevents potential backflow of direct injection or infusion [11] and has been successfully used to deliver compounds in diseases characterised by local pathology including brain tumours and Parkinson’s disease [11, 12]. Cranial navigation software and hardware such as Brainlab (Munich, Germany) [13] can accurately plan trajectories and allow for more precise catheter placement and, in combination with CED, has the possibility to treat diseases with global pathology.

Sheep are an attractive large animal model as their gyrencephalic brain, skull thickness, porosity and the curvature of the calvarium are similar to humans [14]. The sheep brain at maturity weighs around 140g [15], which is larger than most non-human primates and about one tenth of a mature adult human brain.

The present study was designed to determine optimal flow rates for maximal AAV-GFP spread in the unaffected sheep brain using two different CED delivery systems including the Brainlab clinical catheter, and secondly to provide biodistribution data for CED mediated delivery of AAV-HGSNAT.

## Results

### Cranial navigation using Brainlab allows for safe vector delivery in sheep brains and accuracy of target trajectory

**Fig. 1.**
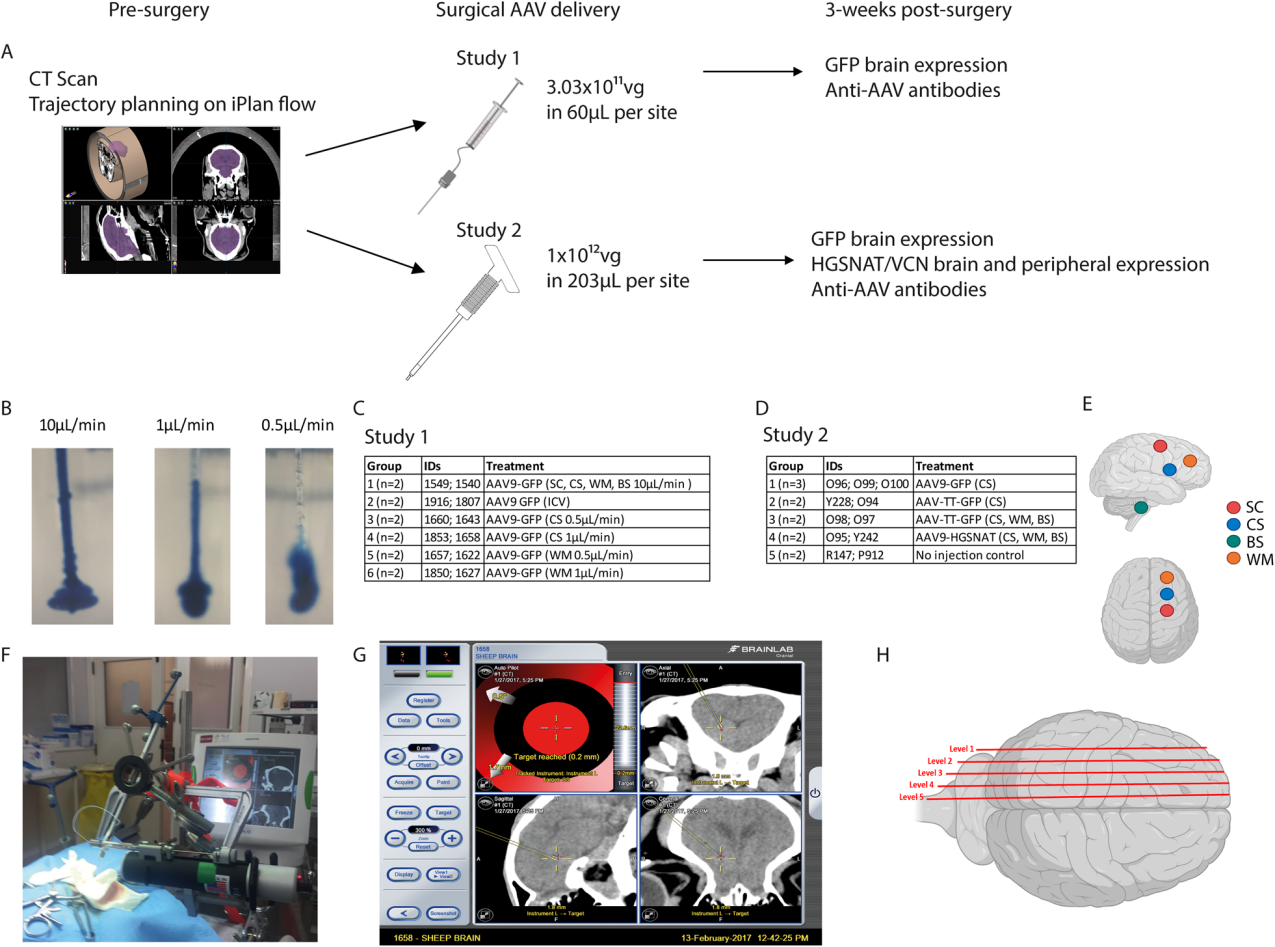
Experimental protocol for AAV distribution studies. **A** Overview of treatment protocol and tissue analysis. **B** Different flow rates in 0.4% agarose gel phantoms. Experimental groups used in study 1 **C** and study 2 **D**. **E** Schematic of injection sites for Corpus Striatum (CS), White Matter (WM), Subcortical zone (SC) and Brainstem (BS) from a lateral and superior view. **F** Syringe driver used for AAV delivery. **G** Example surgical screenshot from Brainlab showing target accuracy. **H** Schematic of mediolateral sagittal brain levels 1-5 used for immunohistochemical analysis

In order to optimise the CED method and to ensure accurate target acquisition in the brain, sheep underwent a pre-surgery CT scan to allow precise mapping of brain vascularisation against skull registration. Target trajectories were planned on the Brainlab iPlan flow cranial navigation system (Fig. 1A) in a similar way to standard clinical surgical trajectory planning. In study 1 we aimed to compare the best target site for injection and the optimal flow rate, whilst using identical injection volume and titre of AAV vector expressing GFP. An AAV titre of 3x10^11^vg/site and injection volume (60 µL/site) were delivered via an intraparenchymal route at each injection site, the exact same intraparenchymal AAV titre and volume used in a clinical trial in MPSIIIA patients [5]. In study 2 we assessed AAV9 vs AAV-TT using a clinical catheter based on the results from study 1 to determine delivery location.

The optimal flow rates for intraparenchymal injections, designed to achieve most efficient vector spread, were determined *in vitro* using gel phantoms, which mimic the density of brain tissue (Fig. 1B). Dye was injected into 0.4% agarose using flow rates of (left to right) 10, 1, 0.5 µL/min were used, with minimal backflow achieved with either 1 or 0.5 µL/min rates. Dye spread was assessed via visual inspection and these rates were determined to be sufficient to compare *in vivo.* We subsequently injected 12 sheep with AAV-GFP comparing flow rates determined from phantoms using either intraparenchymal or intracerebroventricular injections. In study 1, all sheep received 3×10^11^ vg in a total of 60 µL per injection site using target sites to the Corpus Striatum (CS), White matter (WM), Sub-Cortical zone (SC), Brainstem (BS) or Intracerebroventricular to the lateral ventricle (ICV) at flowrates of 0.5, 1 and 10 µL/min (Fig. 1A, C, E). In this study, we used a modified Hamilton syringe to deliver the vector through a custom-made catheter attached to a syringe driver (Fig. 1F).

Study 2 was designed to evaluate methodologies already used in patients such as modality fiducial markers for better registration and clinical catheters in preparation for translation to human application. We first assessed the transduction capabilities of different AAV serotypes in the sheep brain, comparing a serotype (AAV9) that has been effective in previous studies and a novel serotype (AAV-TT) that has only been assessed in preclinical animal studies to date. All sheep were injected with 1x10^12^ vg of AAV-GFP or AAV-HGSNAT in a total of 203 µL per injection site, to the CS, WM and BS, at a flow rate of 1.7 µL/min (Fig. 1A, D, E). The flow rate was slightly modified to accommodate the Brainlab catheter. We compared intraparenchymal injection of GFP using two serotypes: AAV9 or AAV-TT using Brainlab catheters that can be used clinically [13]. In study 2, we also used the AAV-HGSNAT vector to assess biodistribution of this vector using the Brainlab catheter to deliver a therapeutic AAV product for MPSIIIC.

In both studies, surgical navigation via Brainlab was accurate for catheter placement according to pre-planned trajectories on CT scans, however variability within groups suggests that image registration may have been less accurate due to the quality of pre-surgery scans (Fig. 1G). However, we found the use of fiducial markers for digitisation in study 2 made image registration and surface matching easier. Overall, all sheep recovered well post-surgery, however in study 2, P21 from the AAV9-HGSNAT treated group began to develop complications typically associated with a brainstem injury including nystagmus, irregular breathing and limb weakness approximately 2 hours post-surgery. This sheep had a post-operative CT scan and was euthanised due to an evident brainstem bleed, suggesting this was a surgical complication due to catheter placement into an eloquent area and was not caused by the vector. For GFP and HGSNAT analysis, sheep were sacrificed three weeks post-injection and brains cut as described (Fig. 1H).

### Intraparenchymal CED delivery is superior to ICV injection for equivalent volume and titre

**Fig. 2:**
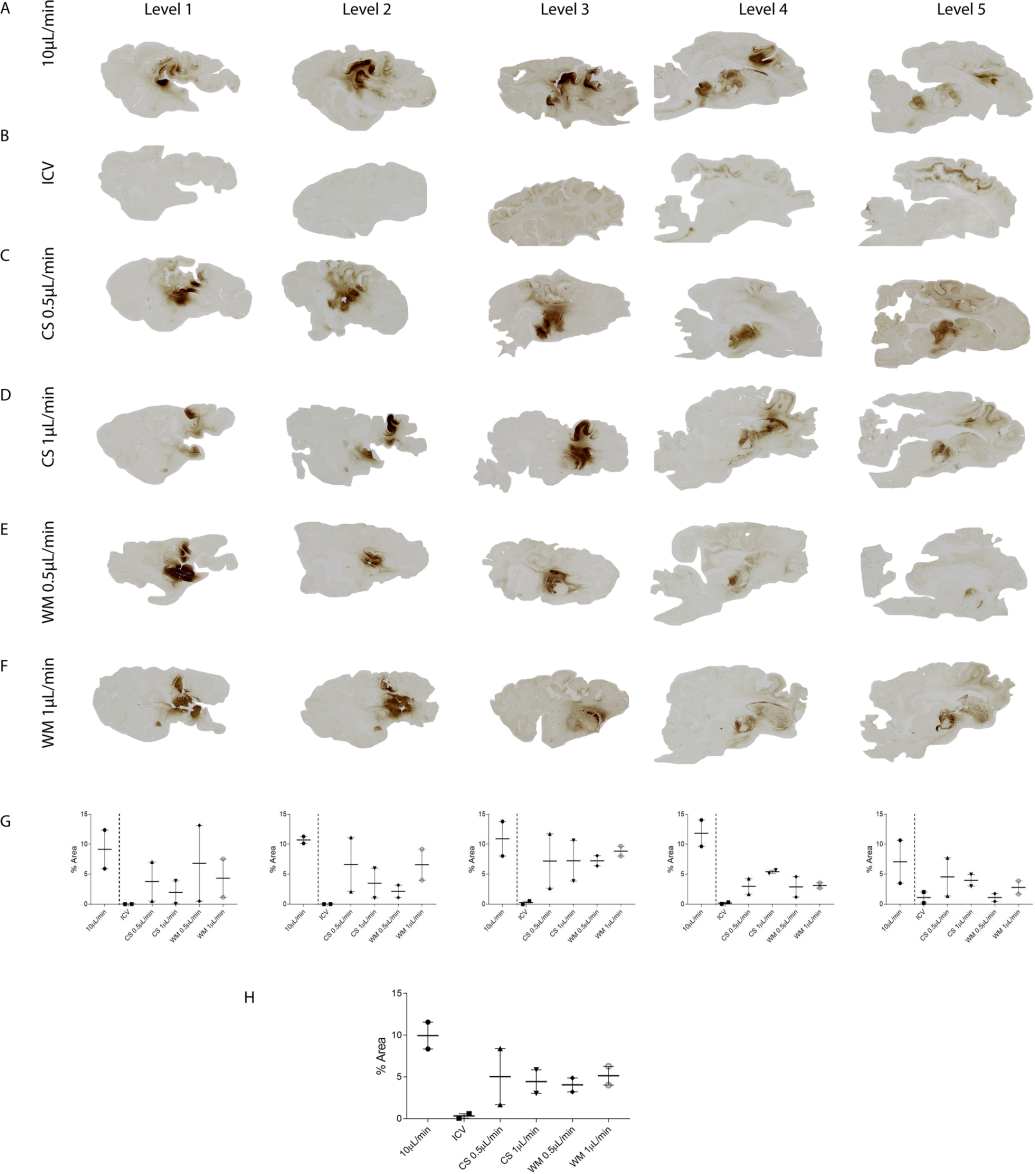
Comparative distribution of GFP expression in sheep brains in study 1. Images showing GFP expression in sheep brains 3 weeks post-injection. Sheep received AAV9-GFP (n=2/group) either into **A** four locations: the corpus striatum (CS), white matter (WM), subcortical zone (SC) and brainstem (BS), **B** the lateral ventricles (ICV), (**C**, **D**) CS and (**E**, **F**) WM with the latter two groups separated into two infusion flow rates (0.5 µL/min and 1 μL/min). **G** Average GFP expression across each sagittal brain level. **H** Average GFP expression across the entire hemisphere. Data are presented as mean ± s.e.m

**Fig. 3:**
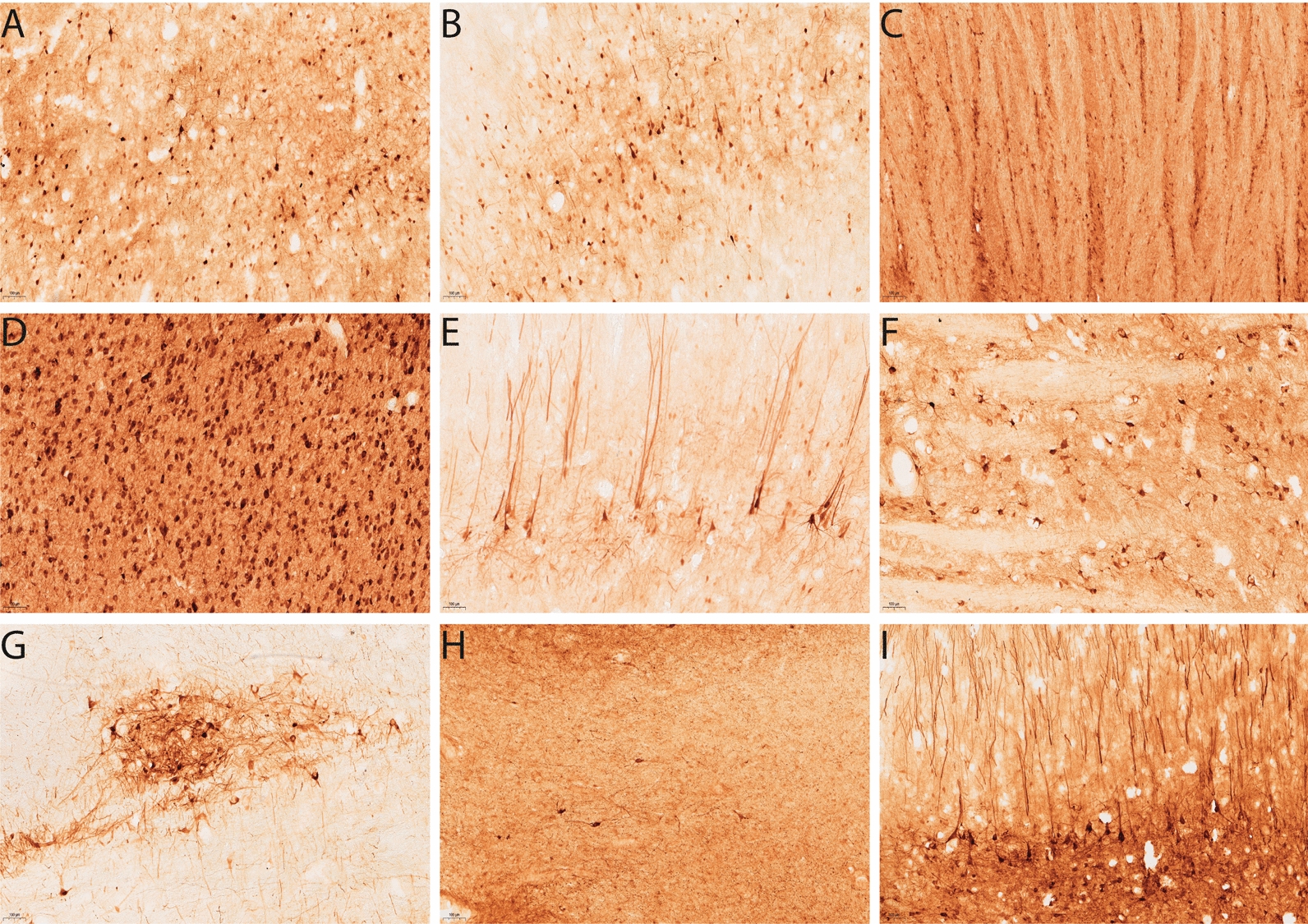
GFP expression from different injection sites in sheep brains in study 1. Images revealing the pattern of GFP expression 3 weeks post AAV9-GFP injection at 10× magnification. After delivery into the lateral ventricles transduced cells can be visualised on the **A** dorsal and **B** ventral surface of the ventricle. GFP transduced cells can be visualised in the **C** striatum, **D** medial geniculate nucleus , **E** motor cortex, **F** brainstem and **G** thalamus after direct injection into the CS. For WM injections, GFP positivity was detected in the **H** WM and the **I** neurons adjacent to the injection site. Scale bar = 100 µm

In study 1, we compared different CED flow rates using the same volume and titre of vector, with either 10 µL/min at 4 different locations in the same brain (the CS, WM, SC and BS) (Fig. 2A) to flow rates of 0.1 µL/min and 0.5 µL/min in single injections into the CS (Fig. 2C, D) or WM (Fig. 2E, F) against the same volume delivered ICV (Fig. 2B), a route commonly used for drug delivery in the brain. Surprisingly, ICV injections performed worse than intraparenchymal injections. Transduced cells were mainly observed on the dorsal and ventral surface of the lateral ventricle (Fig. 3A, B). In the ICV delivery group, very diffuse or no cell transduction was observed at mediolateral sagittal brain levels 1–3, however, both sheep in this group behaved differently to each other. For example, 1916 demonstrated diffuse cortical transduction around the occipital grey matter, in addition to the frontal cortex (Additional file 5: Fig. S5), whilst 1807 had only a small region of focal staining in the corpus callosum (Additional file 5: Fig. S5).

Of the different intraparenchymal injections, those delivered at 4 different target sites gave the highest overall level of transduction, consistent with the higher total vg dose delivered. In these sheep, there was diffuse spread around the thalamus and striatum which extended to the white matter from the injection site in brain levels 3 and 4. No staining was observed on the contralateral side of the brain in the sheep that received single injections. More localised transduction can be observed in the subcortical regions that extend laterally through levels 1-5 and ventral GFP transduction through the brainstem in level 4 and 5 (Fig. 2A). This group was excluded from our comparative analysis as four doses of vector had been injected. Average GFP expression across each sagittal brain level was assessed (Fig. 2G) in addition to average GFP expression across the entire hemisphere (Fig. 2H).

Different CED flow rates in single intraparenchymal injections produced similar levels of expression even though the location of their target brain areas was different. For CS injections at 0.5 and 1 µL/min, transduction remained mostly in areas close to the injection site in the striatum (Fig. 3C), the medial geniculate nucleus (Fig. 3D), the thalamus (Fig. 3G), with some pyramidal neurons evident in the motor cortex (Fig. 3C–E). Interestingly, transduced cells could be observed in the brainstem in one sheep from the CS 1 µL/min (Fig. 3F) group. A more localised pattern was observed with the WM injections (Fig. 3H, I), although any transduced cells observed at distant sites may be due to retrograde transport of vector. The vast majority of cells transduced by these routes had the appearance of neurons.

However, when we examined the average expression across the entire hemisphere in study 1, all CED delivery methods produced similar results; with ICV delivery performing the worst and resulting in an average of 0.32% of the brain hemisphere transduced (Fig. 2H). There were significant differences (p<0.05) between ICV and either CS 0.5 µL/min (5.0%); CS 1 µL/min (4.4%) or WM 0.5 µL/min (4.4%) despite equal viral titres and vector volumes injected. No significant differences in GFP expression were observed between white and grey matter injections, with approximately 4.5–5% of a hemisphere expressing GFP from a single injection site.

Overall, with 4 injections sites and using a 10 µL/min flow rate (4 times more vector than the single site injections), we achieved an average of approximately 10% hemisphere transduction, compared to 4.5–5% with single injections, suggesting that either there was some overlap between cells transduced in the single injections (Fig. 2H), but perhaps that this flow rate may reduce overall spread of vector.

### Catheter design can affect vector distribution to the brain

In preparation for translation to human application, study 2 used currently approved clinical catheters (Brainlab) [13]. Although an independent study, study 1 data was used as an indirect comparator to determine if specialised stepped catheters could provide better vector spread than our original custom-made catheters. Following the outcome of study 1, we removed the ICV group from study 2, and the CS was chosen as the comparative target region because spread here was more consistent between individual sheep and also may be more desirable for clinical application. In this study, we slightly adjusted the flow rate to 1.7 µL/min to accommodate the volume required for the clinical catheter. Unpredictably, even though we increased the vector amount and volume delivered was substantially greater, to 1×10^12^ vg/site in 203 µL, the percentage of area transduced per brain was less than that of study 1.

### AAV-TT displays a trend towards better transduction compared to AAV9 in the sheep brain

**Fig. 4:**
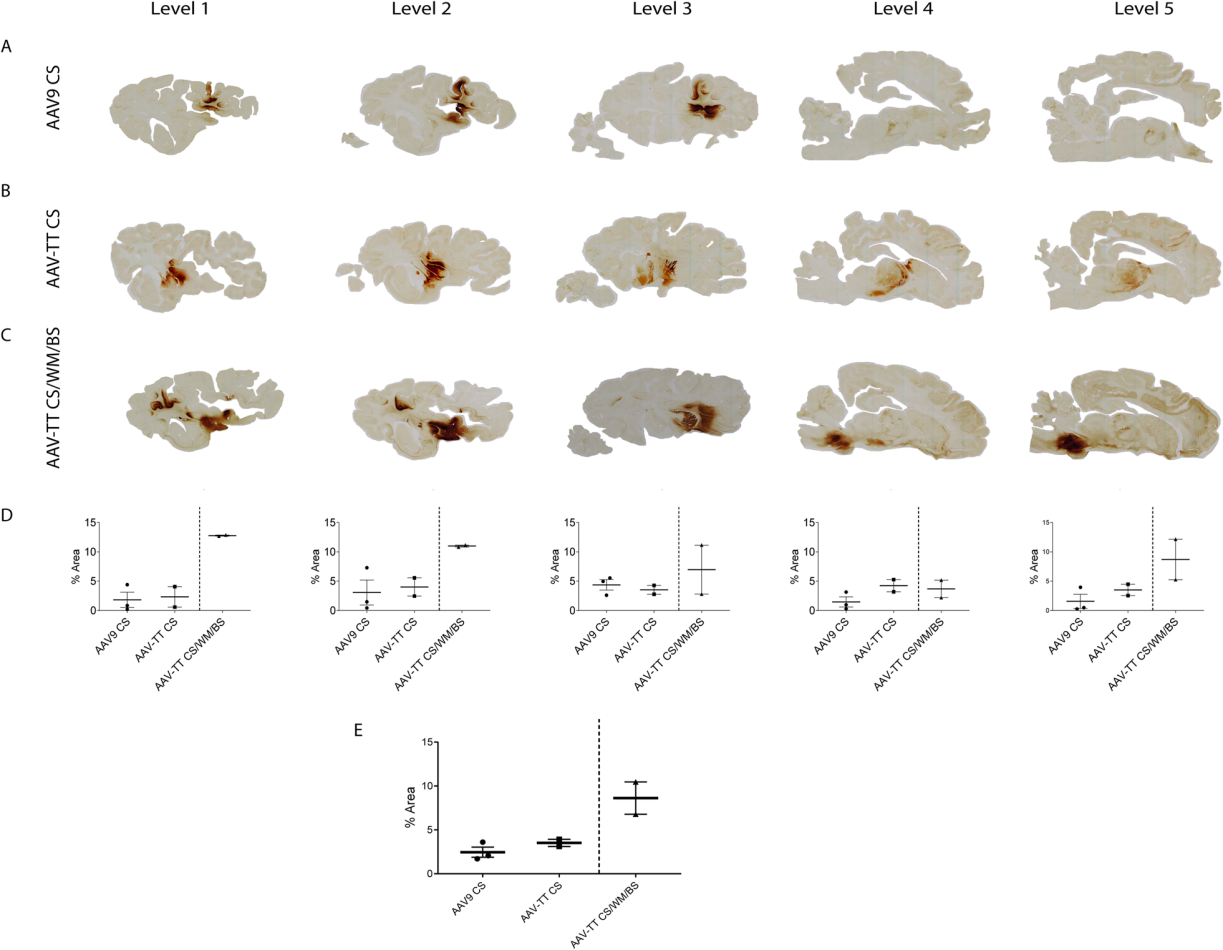
Comparative distribution of GFP expression in sheep brains in study 2. Images showing GFP expression in sheep brains 3 weeks post-injection. Two groups received either **A** AAV9-GFP (n=3) or **B** AAV-TT GFP (n=2) into the corpus striatum (CS) only. **C** An additional group of sheep (n=2) received AAV-TT-GFP into the CS, white matter (WM) and brainstem (BS). **D** Average GFP expression across each sagittal brain level. **E** Average GFP expression across the entire hemisphere. Data are presented as mean ± s.e.m

**Fig. 5:**
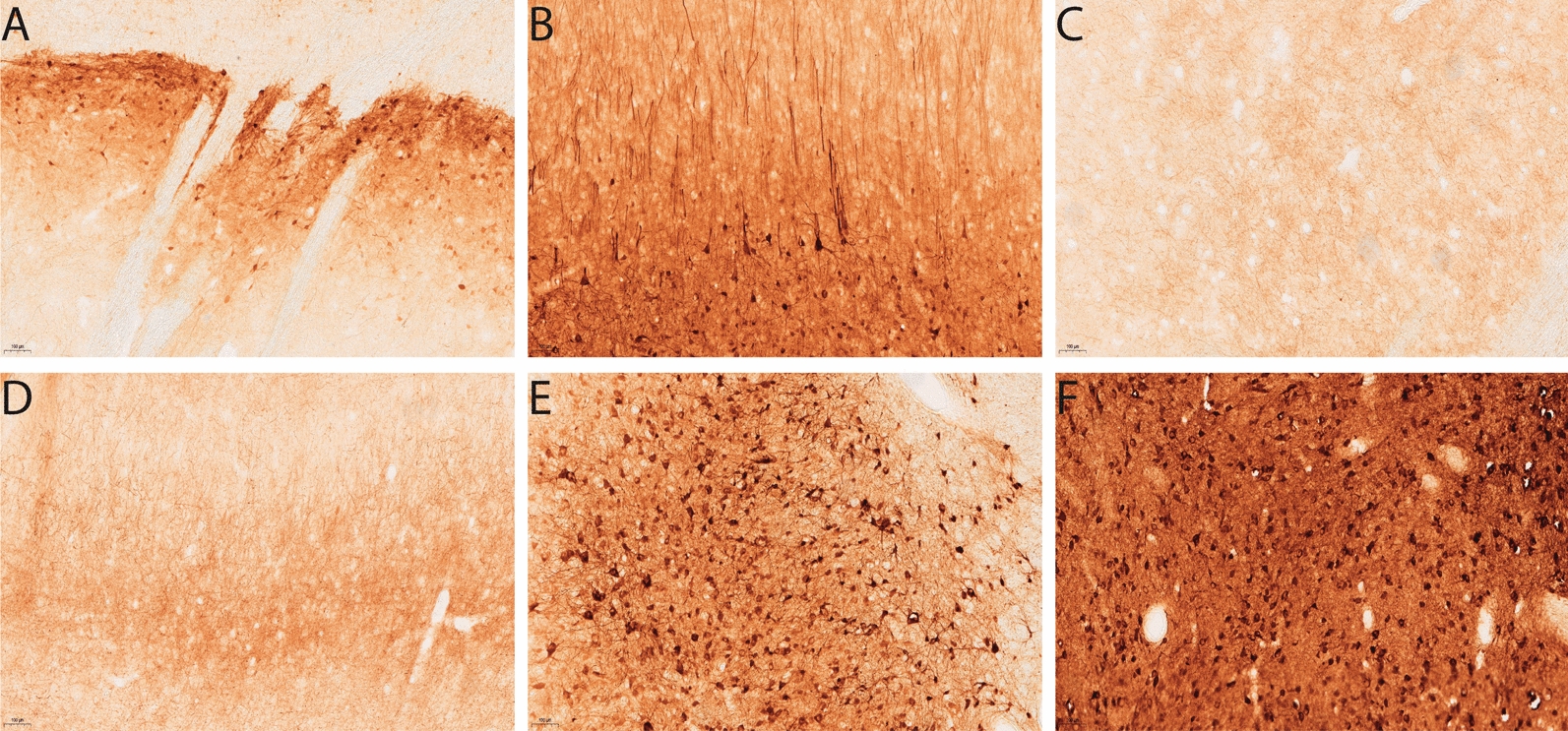
GFP expression from serotypes in sheep brains in study 2. Images revealing the pattern of GFP expression 3 weeks post AAV-GFP injection at 10× magnification. AAV9-GFP injection into the corpus striatum (CS) displays GFP transduced cells in the **A** striatum and **B** motor cortex. AAV-TT-GFP transduced cells can be visualised in the **C** striatum, **D** motor cortex, **E** medial geniculate nucleus and **F** thalamus. Scale bar = 100 µm

In study 2, to determine the most efficient serotype for brain distribution, the expression levels of AAV9 and AAV-TT vectors containing a GFP transgene were compared. Distribution was assessed via immunohistochemistry for GFP positive cells. From a single injection site, AAV-TT gave more diffuse vector spread despite delivering equivalent vector titres and volumes. In 4/5 of the assigned mediolateral sagittal brain levels, AAV-TT displayed a trend towards higher transduction compared to AAV9; Level 1 AAV-TT 2.3% vs AAV9 1.8%; Level 2 AAV-TT 4.0% vs AAV9 3.1%; Level 3 AAV-TT 3.5%; vs AAV9 4.4%; Level 4 AAV-TT 4.2% vs AAV9 1.5%; Level 5 AAV-TT 3.5% vs AAV9 1.6% (Fig. 4A, B, D). Expression from AAV9 CS injections mainly localised around the site with some transmission into the motor cortex (Fig. 5A, B) and whereas AAV-TT albeit less intense extended into the medial geniculate nucleus and putamen (Fig. 5C–F). AAV-TT was however not significantly better than AAV9 in terms of overall percentage of the hemisphere expressing GFP, 3.5% (AAV-TT) vs 2.5% (AAV9) (Fig. 4E) likely due to the low n numbers used.

These data are consistent with previous studies showing that AAV9 produces stronger localised transduction near the injection site compared to AAV-TT which results in a better overall distribution [3].

Notably, again as in study 1, three injections of AAV-TT produced a total percentage transduction of around 8.6% of brain hemispheres vs 3.5% for the single injections, suggesting there may be some overlap between cells transduced per site (Fig. 4C, E).

### AAV9 and AAV-TT specifically transduce neurons in the sheep brain

**Fig. 6:**
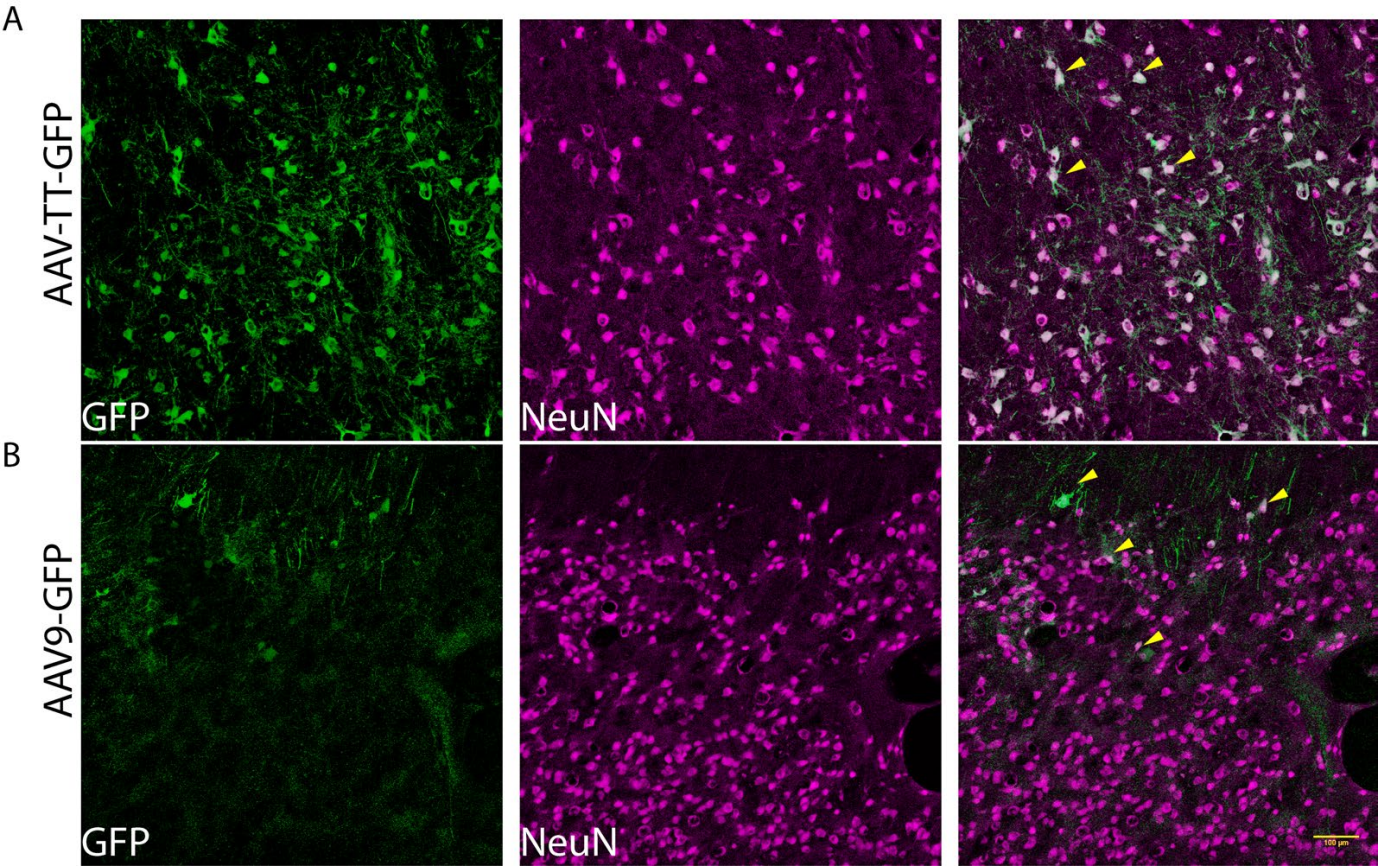
AAV vectors target neurons in the corpus striatum of the sheep brain. Double staining of GFP+ (green) with NeuN+ neurons (magenta) in **A** AAV-TT-GFP and **B** AAV9-GFP treated sheep at 10× magnification. Arrows in the merged image indicate examples of co-localization of GFP+ and NeuN+ cells. Scale bar = 100 µm

As seen from GFP distribution studies, the majority of cells transduced with either serotype had the appearance of neurons (Fig. 5). However, to clearly determine which cells were transduced by the different vectors, double-labelling of GFP+/NeuN+ cells in the CS was analysed by immunofluorescence microscopy. GFP expression was exclusively co-localised in neurons (Fig. 6). Consistent with our observation in rodents [3], no GFP was detected in either GFAP+ astrocytes or Iba1+ microglia/macrophages (Additional file 11: Fig. S11).

### Pre-existing anti-AAV antibodies do not appear to affect AAV transduction in the sheep brain

**Fig. 7:**
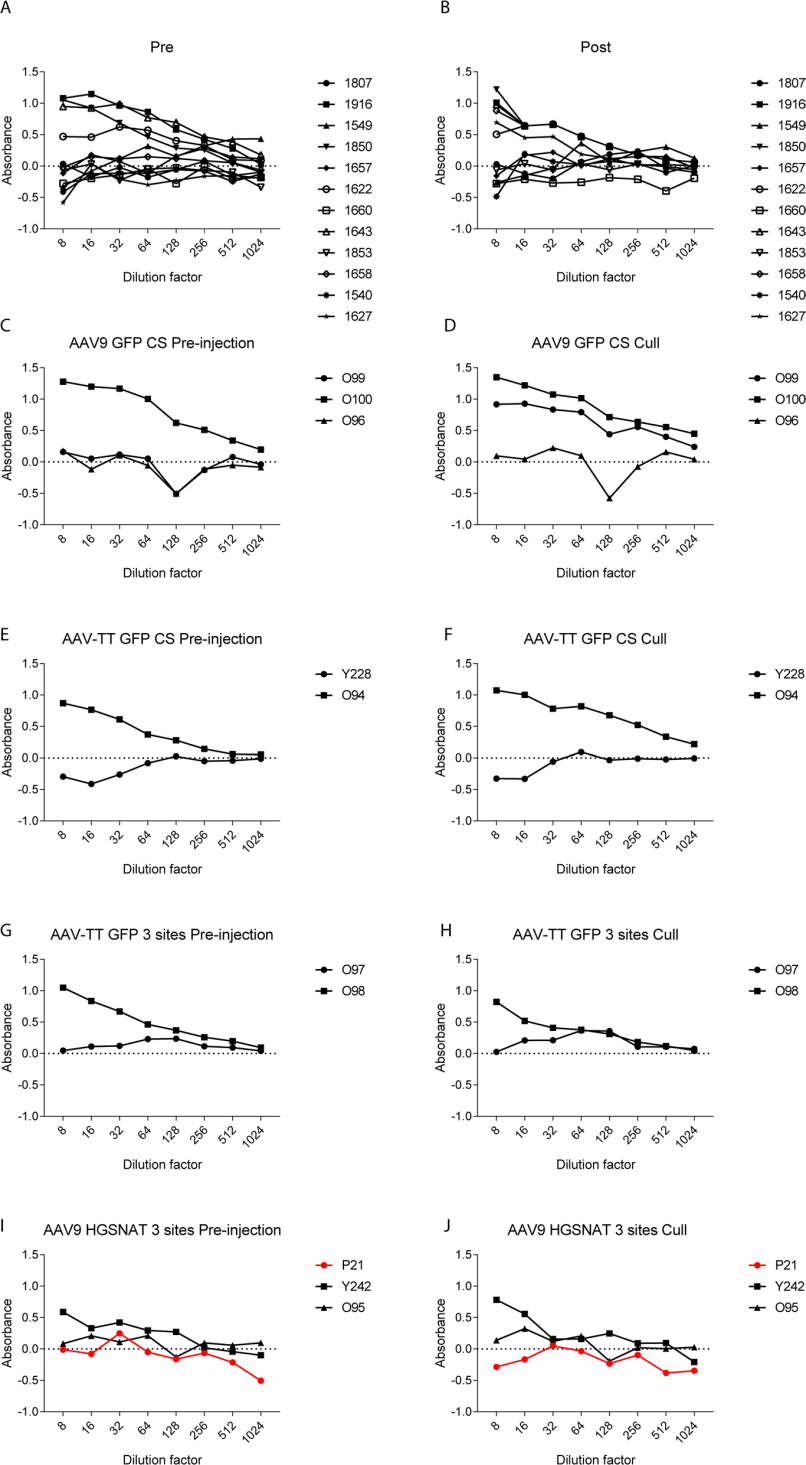
Immune reactions pre- and post-injection. Total IgG antibody responses against AAV9 and AAV-TT capsid proteins measured by ELISA on day 0 (the day of surgery) and 3 weeks post-surgery (at necropsy) in study 1 (**A**, **B**) and study 2 (**C**–**J**). A number of sheep showed pre-existing antibodies to the AAV-capsids that were still evident at necropsy. **D** One sheep developed an immune response to the AAV capsid. **J** One sheep (highlighted in red) was sacrificed on the day of surgery due to an adverse event but had no IgG antibodies against the capsid

**Fig. 8:**
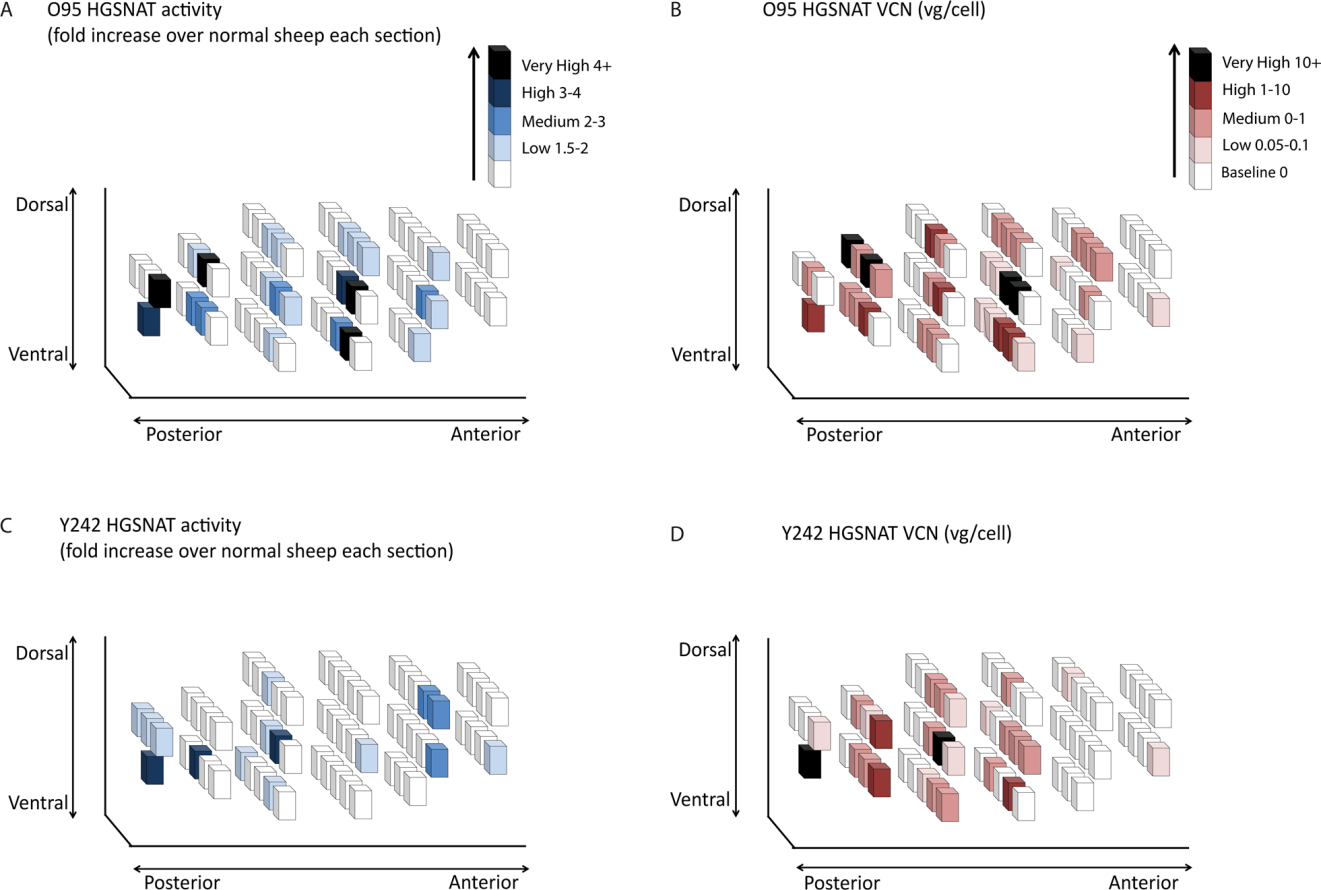
Comparison of HGSNAT expression in AAV9-HGSNAT-treated sheep 3 weeks post-surgery. Vector distribution is represented by three-dimensional schematic drawings of the brain. Each brain was divided into 1cm^3^ cubes. Quantitative comparison of HGSNAT activity above endogenous background level was assessed (**A**, **C**). HGSNAT values were determined as fold increase compared to the mean of 2 un-injected sheep for that relative brain section and VCN in two different sheep O95 and Y242 (**C**, **D**)

As the existence of systemic anti-AAV immunity may dampen AAV expression, the serum of all treated sheep was assessed for the development of total IgG related anti-AAV antibodies both before and 3 weeks post-surgery. In both studies, a number of sheep, approximately 33% for study 1 (Fig. 7A, B) and 40% for study 2 (Fig. 7C–J), had pre-existing antibodies to either AAV9-GFP (1916, 1643, 1622, 1850, O100), AAV-TT-GFP (Y228, O98) or AAV9-HGSNAT (Y242). The presence of pre-existing antibodies in peripheral blood did not appear to correlate with better or worse injection outcomes in the brain (Fig. 8).

Even though in both studies some sheep developed antibodies against AAV9-GFP (1658, 1627, O100) 3 weeks after surgery (Fig. 7B, D), we still observed GFP expression in the brain (Additional files 5, 6, 7, 8, 9, 10: Figs. S5–S10).

### AAV9-HGSNAT delivery at three sites in one hemisphere results in HGSNAT activity in 24-37% of the sheep brain using Brainlab catheters

To determine the spread of HGSNAT enzyme expression around injection sites in the brain, HGSNAT expression was assessed in 4 sheep (2 un-injected controls and 2 AAV injected). These sheep were injected with AAV9-HGSNAT into 3 areas in one brain hemisphere, the CS, WM and BS, all concurrently, at 1.7 μL/min flow rates to mimic the clinical delivery approach. Three weeks later, the brain was divided in 1cm^3^ cubes (anterior to posterior; A–F, Fig. 8). HGSNAT activity and vector copy number (VCN) levels were measured in each 1cm^3^ cube to determine vector distribution, using a uniform grid as a reference. Each sheep brain contained approximately 63 cubes. The increase in HGSNAT activity over endogenous baseline in the 1cm^3^ brain cubes is presented as a 3 dimensional plot of the cubes assembled in a brain matrix. HGSNAT values were determined as fold increase compared to the mean of 2 un-injected and unaffected sheep for that relative brain section. Increases in enzyme activity were defined as baseline (0-1.5), low (1.5-2), medium (2-3), high (3-4) and very high (4+) fold over normal HGSNAT activity. Overall, we see HGSNAT activity above baseline for sheep O95 in 23 cubes or 36.5% of the brain (Fig. 8A) and this expression varied in different brain cubes ranging from baseline (63.5%) low (19%), medium (7.9%), high (3.2%), and very high (6.3%). A similar pattern albeit lower expression pattern was observed for sheep Y242 with enzyme activity in 15 cubes or 23.8% of the brain (Fig. 8C), again ranging from baseline (76.2%), low (14.3%), medium (4.8%) and high (4.8%).

VCN was also assessed in each brain cube via qPCR using primers specific for the AAV-HGSNAT vector. AAV vg/cell expression was classified as baseline (0-0.05), low (0.05-0.1), medium (0.1-1), high (1-10) and very high (10+) fold HGSNAT expression over normal. A comparable result was observed for VCN in sheep O95 with 34 cubes or 53.9% of the brain expressing HGSNAT (Fig. 8B), again each cube varied from baseline (46%), low (12.7%), medium (25.4%), high (9.5%) and very high (6.3%). Interestingly, sheep Y242 which had pre-existing antibodies gave poorer results, with fewer high and very high cubes. However, Y242 still had VCN expression above baseline in 26 cubes or 41.3% of the brain expressing HGSNAT (Fig. 6D), baseline (58.7%), low (12.7%), medium (20.6%), high (4.8%) and very high (3.2%).

This spatial representation, from only 3 injections in a single brain hemisphere demonstrates that widespread distribution can be achieved with CED.

### Little off-target expression is observed in AAV-HGSNAT treated sheep

Fifteen peripheral organs were processed and analysed for HGSNAT activity and VCN to determine off target vector distribution. Two of the AAV9 treated sheep had two inflamed mesenteric lymph nodes, which were taken for further analysis. There was no evidence of elevated HGSNAT activity or detectable VCN in the lymph nodes tissues. We found no differences in HGSNAT activity between AAV injected and un-injected controls in any other organs sampled including three different regions of spinal cord, CSF, bone marrow, pancreas, spleen, liver, lung, kidneys, ovaries, trachea, oesophagus, small intestine, bladder, lymph node and heart (Additional file 12: Fig. S12).

Consistent with enzyme activity, very little or no VCN levels were detected in most organs (Additional file 13: Table S1), the highest VCN level was observed in the pancreas at 0.25 vg/cell, suggesting little to no off target delivery of HGSNAT using the CED approach.

## Discussion

The main aim of these studies was to determine an optimal delivery technique for direct brain injection of AAV for diseases with global pathology such as MPSIIIC. Although MPSIIIC is a lysosomal disease, the enzyme is transmembrane and not secreted, making it difficult to achieve adequate enzyme levels in the brain to correct the disease following gene therapy. Therefore, optimal delivery to cells throughout the brain either by improved surgical techniques, and/or advances in vector technology are required. Direct intracranial injection volumes are limited by the risk of oedema, and multiple injections are typically required to adequately cover the whole brain in a human patient [16]. The ICV route may prove an issue due to the presence of the blood/CSF barrier preventing sufficient vector diffusing from the CSF into the brain parenchyma [17]. Intracisterna magna injection limits vector spread to the areas caudal to the brain, but currently is rarely used in humans in preference to the intraventricular route because of the risks involved with surgery in that area [18]. Both techniques have been reported to result in predominantly glial cell transduction rather than neuronal transduction [8] and both require relatively large amounts of vector to achieve efficient delivery compared to intraparenchymal approaches.

Although the two studies used different volumes and titres of vector, and as such are not directly comparable, we observed that despite higher titre and volume to the CS site using Brainlab catheters vector spread was lower. One possible reason for the discrepancy is that study 2 used FPLC (affinity) purified vector in preparation for clinical trial, whilst study 1 used iodixanol gradient purification. The full:empty capsid ratio or post-translational modification of the capsid may also be responsible for this. The use of a Brainlab catheter appeared to be less effective than a Hamilton syringe. One explanation is that catheters originally designed for delivery to brain tumours are not optimal for gene therapy delivery via CED. If this is the case, then it may apply to almost every catheter available today. A number of preclinical studies have shown that a single injection of AAV directly into the CSF can achieve widespread distribution of vector through the CNS [19–21]. Various AAV vectors have been tested via intra-CSF delivery with different reports of outcomes in preclinical models [22–26], with AAV9 proving to be most promising. In contrast to a previous study in sheep that achieved widespread distribution after ICV administration [27], we found that intra-CSF delivery into the ventricles resulted in vector transduction limited to cells at the perimeter of the ventricle with minimal spread to brain structures. Understandably, our aim was to deliver a much lower volume and titre to be able to directly compare to an intraparenchymal injection, nevertheless we achieved 5% brain transduction with an intraparenchymal injection of 3×10^11^vg in a total of 60 µL per ventricle in study 1, whilst this was almost ineffectual in our hands in ICV delivery. Mitchell et al required over 10 times more vector (4×10^12^ vg in a total of 400 µL per ventricle) to achieve effective brain transduction. An infusion volume of 60 µL may not be sufficient to observe robust distribution from the ICV delivery, it may cause the vector to be diluted in the 2–4 mL of circulating CSF of the sheep brain. The greater infusion volume of 400 µL could be attributed to the better transduction observed in Mitchell et al. We hypothesise that the major difference is the achieved flow rate and intraventricular pressure, and if optimised this could reduce vector requirements significantly whilst achieving effective delivery to the brain. AAV treatment in a sheep model of Tay Sachs disease led to delayed symptom onset and disease progression with enhanced quality of life [28] using a combination of both intraparenchymal and ICV delivery. This may be possible, however, large amounts of vector would be required. A trial of human heparan-N-sulfatase (rhHNS) for MPSIIIA administered via an intrathecal drug delivery device reported failures with the delivery method, suggesting that repeated delivery regime is not ideal [29]. In addition, a phase I-II trial is currently enrolling patients for AAV9 ICV delivery in MPSIIIA patients (EudraCT 2015-000359-26).

The specific area within the brain that was targeted did not appear to affect distribution of vector; typically, we achieved approximately 4-5% hemisphere coverage per injection in each study. Higher flow rates (10 µL/min), were associated with less effective overall coverage of the brain than either 0.5 or 1 µL/min flow rates (4 injections: 10% coverage vs 1 injection: 4-5% coverage). Some brain areas transduced may overlap but this should not account for a 50% difference.

Overall, the vectors and delivery methods were well tolerated across both studies. However, in study 2 one sheep developed complications which were associated with brainstem injury as a result of catheter placement, with a bleed evident on CT scan and at necropsy. This sheep also had a movement artefact on the pre-operative planning CT scan that may well have affected catheter placement accuracy. Pre-existing total IgG antibodies existed in the sera of some sheep, however, gene expression was still observed in the brains of these sheep. One or two of these showed notably lower spread of vector compared to their counterparts which could be an inhibitory effect. Only one sheep in study 2 developed antibodies against the AAV9-GFP vector, and immunoreactivity against GFP has been observed previously as a reaction against the foreign protein [23]. No anti-AAV HGSNAT antibodies were detected in the serum of sheep which had been administered the vector containing the human form of HGSNAT.

AAV tropism, based on different serotypes, is another important consideration affecting gene therapy efficiency. As previously described [3], we found that both AAV9 and AAV-TT appeared to specifically transduce neurons in the sheep brain when delivered via convection enhanced delivery into brain parenchyma. For our therapeutic vector, we chose AAV9 as it has already been used effectively in clinical trials.

We observed effective brain delivery from only three concurrent CED injections into one brain hemisphere, which resulted in 24-37% brain transduction assessed via HGSNAT activity but 41-54% brain transduction assessed via VCN. This demonstrates that catheters with skull fixations can effectively be used to deliver AAV to a large animal brain. As we only delivered vector to half the brain with this technique, achieving HGSNAT activity of two-fold or greater than normal across 24-37% of the entire brain is a significant achievement and may be sufficient to move forward to clinical trial. The number of brain cubes with HGSNAT levels above baseline was less than that of VCN containing cubes. One reason for this may be because these sheep have a functional HGSNAT gene which produces enzyme, meaning that small subtle differences in expression may be undetectedor it could be due to the presence of empty AAV capsids. Minimal off-target effects were observed when biodistribution of the vector was examined, this may be explained by the presence of antibodies in the sera of some sheep. At necropsy, an inflamed mesenteric lymph node was found in both the AAV9-HGSNAT-treated sheep however, no enzyme or VCN were detected, suggesting it was not vector related.

The value of catheter design is evident from the data presented and shows that small differences can affect overall spread of vector. Surprisingly, the clinically approved Brainlab catheter, even with increased titre and volume, resulted in less GFP expression than our in-house, custom-made catheter. Interestingly, we observed less variability between sheep in study 2 which we hypothesise was due to the addition of fiducials allowing for easy surface matching and patient registration. In study 2, the overall transduction values across the hemisphere were less than those in study 1 suggesting that this commercial catheter design could be suboptimal for maximum CED based vector distribution.

The Brainlab system was originally designed for localised drug delivery and surgical resection of brain tumours, not global gene therapy. Thus, catheter tip profiles may affect vector spread; the custom-made catheter having an end-point, sharp cannula and the Brainlab catheter comprising of a stepped profile with a blunt tip [30], which in turn may affect the pressure required for fluid distribution in the brain. Our custom-made catheter had a smaller internal diameter of 0.41 mm and an external diameter of 0.718mm diameter. The tip of the Brainlab catheter had an internal diameter of 0.536mm and an external diameter of 0.66mm, however the majority of this catheter was 2.08mm. One possible reason for the poorer distribution observed with the Brainlab catheter could be due to reflux caused by a wider external catheter diameter as larger dimensions of catheters are prone to reflux [28]. Of course, the higher titre and volume of vector could also have been a factor in this.

## Conclusion

In summary, our clinical catheter outcomes suggest that optimisation of flow rate and catheter design are both parameters that could be further adjusted to improve overall delivery outcomes via CED. The use of adjuvant substances such as mannitol, systemically administered may facilitate AAV distribution even further [31]. Critically, flow rate and pressure gradients are probably the more important factors in scaling from large animals upwards, not titre and volume.

Finally, we have successfully delivered AAV HGSNAT vector to the brains of sheep achieving up to 36% distribution in a sheep brain from 3 CED based injections in a single hemisphere, with little off target delivery of vector, therefore paving the way for future clinical trials.

## Material and methods

### Animals

Female 6 month old Coopworth sheep *(Ovis aries)* were maintained at Lincoln University, Lincoln, New Zealand under NIH guidelines for the care and use of animals in research and the New Zealand Animal Welfare Act (1999).

### Study design

Two independent studies were completed. In order to determine distribution throughout the brain with no overlap, 4 anatomical and functionally important distinct regions were chosen. The locations chosen for AAV injection were the corpus striatum (CS) and white matter (WM), subcortical zone (SC) and brainstem (BS). Previous studies have shown these locations, particularly the brainstem to be safe targets[32]. All sheep were sacrificed at 3 weeks post-surgery.

#### Study 1

All sheep received 3 x10^11^vg in a total volume of 60 µL per injection site. All vectors were under the control of the ubiquitous chicken beta actin/CMV composite promotor (CAG) promotor. The experimental groups were Group 1 (n=2): AAV9-GFP (SC, CS, WM, BS 10µL/min), Group 2 (n=2): AAV9-GFP (ICV), Group 3 (n=2): AAV9-GFP (CS 0.5 µL/min), Group 4 (n=2): AAV9-GFP (CS 1 µL/min), Group 5 (n=2): AAV9-GFP (WM 0.5 µL/min), Group 6 (n=2): AAV9-GFP (WM 1 µL/min).

#### Study 2

All sheep were injected with 1×10^12^ vg in a total volume of 203 µL per injection site, to a number of sites with a flow rate of 1.7 µL/min. All vectors were under the control of the ubiquitous CAG promotor. The experimental groups were Group 1 (n=3): AAV9-GFP (CS), Group 2 (n=2): AAV-TT-GFP (CS), Group 3 (n=2): AAV-TT-GFP (CS, WM, BS), Group 4 (n=2): AAV9-HGSNAT (CS, WM, BS), Group 5 (n=2): no injection control.

### Recombinant AAV production

Recombinant single stranded AAV vectors were produced, purified and titered using standard procedures as previously described [3]. Plasmids encoding either codon optimised human HGSNAT (coHGSNAT) or green fluorescent protein (GFP) under the control of the cytomegalovirus (CMV) enhancer fused to the chicken beta‐actin promoter (CAG) were used for AAV production. Recombinant AAV virions were produced by transient transfection of HEK 293T cells using polyethylenimine (PEImax, Polysciences Inc. Hirschberg an der Bergstrasse, Germany) with two plasmids: one plasmid encoding the transgene cassette flanked by ITRs of AAV2 (AAV-CAG-coHGSNAT or pTRUF-11 (ATCC, MBA-331) and the pDG plasmid expressing AAV2 Rep and either the AAV9 or AAV-TT Cap gene, and adenovirus 5 helper functions necessary for virion formation. Cells were harvested 72 hours post transfection; crude cell lysate was produced by repeated freeze/thawing of the cell pellets in lysis buffer to release the virus. In parallel, the virus-containing supernatant was harvested and precipitated using ammonium sulphate salt. The cell lysate and precipitated supernatant were treated with benzonase to digest cellular and non-encapsidated DNA, clarified by centrifugation and filtered at 0.22 µm before purification. For study 1, AAV9 vector was purified by iodixanol step gradient and study 2, AAV9 and AAV-TT virus preparations were purified using the AKTA purifier chromatography system. We performed real time PCR and alkaline gel electrophoresis to assess the viral genome titres and integrity of the viral genome, the capsid titres were determined by SDS PAGE electrophoresis. The vectors were titre matched in PBS before injection.

### Computed tomography and trajectory planning

Computed tomography was conducted using a GE Prospeed CT scanner (GE Healthcare, Hyogo, Japan) as previously described [33]. A pre-operative CT scan was acquired and used as a reference for trajectory planning on iPlan Flow (Brainlab, Munich, Germany). All images and trajectory plans were checked by two neurosurgeons prior to surgery.

### In vivo injections

Sheep were anaesthetised with a single i.v. injection with a mixture of equal volumes of diazepam (0.5 mg/kg live weight;Pamlin injection, Troy Laboratories NZ Pty Ltd, Auckland, NZ) and ketamine (10 mg/kg live weight;Phoenix Ketamine injection, Phoenix Pharm Distributors Ltd, Auckland, NZ). The sheep was placed in the sternal recumbency or prone position. In study 2, fiducial markers were placed on the face to be used as a surrogate for target positioning during registration.

The head was positioned in a stereotactic frame (David Kopf instruments, California, USA), the mouth placed on the horizontal bar of the surgical guide frame and the ear bars inserted into each auditory meatus to hold the head. Anaesthesia was maintained by isoflurane inhalation (2–4% v/v).

ICV injections were performed as previously described [27]. Sheep received bilateral injections into the lateral cerebral ventricles. Briefly, a 3 mm hand drill was used to create burr holes in the skull and an empty Hamilton syringe with a 26G needle was positioned above the brain. The syringe was then filled with sterile saline solution and immediately lowered stereotaxically into the brain. The meniscus level stopped dropping when the needle entered the brain tissue. The needle was then lowered until the level of the saline again began to fall indicating the needle tip was within the lateral cerebral ventricle. The saline was removed from the Hamilton syringe and replaced with the required volume of vector. A sterile plunger was inserted in the Hamilton syringe and the virus injected. Once the complete dose was deposited the needle was slowly withdrawn.

All CED procedures were performed using the Brainlab Varioguide and the Kolibri Brainlab cranial navigation system. Surface matching on the sheep head using the Varioguide was used for image registration. The head was shaved and an incision made. The drill guide was placed through the Varioguide and advanced to the skin surface. A 5.8mm hand drill was used to create a burr hole in the skull.

CED procedures were performed using a custom made catheter for study 1 or a Brainlab catheter system [13] for study 2 (Fig. 1A). The appropriate catheter for each study was placed manually through the burr hole ensuring the intended trajectory was maintained through the Varioguide. In study 1 the tip of the catheter remained in place. In study 2 the catheter was secured to a bone anchor and the stylet removed.

For each study, the catheter was attached to a Hamilton syringe connected to a rate-controlled microinfusion pump (World Precision Instruments, Hertfordshire, UK). CED procedures were performed at a number of infusion rates depending on the study design. After infusion the catheter were left *in situ* for 5 minutes to avoid reflux. The galea was sutured with 3-0 cat-gut and skin with 3-0 Ethilon (Scientific Laboratory Supplies Ltd, Nottingham, UK). Analgesia and sub-cutaneous antibiotics were administered and the sheep monitored until they made a full recovery.

### Tissue collection and preparation

Three weeks after AAV administration, animals were sacrificed and brains perfusion fixed post-mortem via the carotid artery with 10% formalin in 0.9% NaCl.

Brains from GFP-treated sheep were post-fixed in 10% formalin for five days, stored in cryoprotectant (20% sucrose and 10% ethylene glycol in 0.9% NaCl) for five days, and frozen at −80 °C. Serial sagittal brain sections (50 μm) cut on a freezing sliding microtome were stored in cryoprotectant in 96-well plates at −20°C. Mediolateral levels were assigned as previously described [34].

Brains and organs from HGSNAT-treated sheep were snap-frozen on dry ice. Brains that had been snap-frozen brains were dissected on ice into 1cm^3^ cubes prior to processing.

### GFP immunohistochemistry

Endogenous peroxidase activity was depleted by incubating sections in 1% H_2_O_2_ in tris-buffered saline (TBS) for 30 minutes and washing in PBS. Endogenous non-specific protein binding was blocked by incubation in 15% normal goat serum (NGS; Sigma-Aldrich, Dorset, UK) in TBS-T (TBS with 0.01% Triton X-100) for 30 minutes. Sections were incubated overnight in 10% NGS in TBS-T with rabbit anti-GFP (1:10,000-1:20,000 ab290, Abcam, Cambridge, UK). Following washes in TBS, sections were incubated in 10% NGS in TBS-T with biotinylated secondary antibody goat anti-rabbit IgG (1:1,000, B7389, Sigma-Aldrich) for 2 hours, followed by ExtrAvidin peroxidase (1:1,000, E2886, Sigma-Aldrich, Dorset, UK) for 4 hours. Staining was visualized using 0.5 mg/mL 3, 3′-diaminobenzidine (D5637, Sigma-Aldrich, Dorset, UK) and 0.01% H_2_O_2_ in PBS, after which the sections were mounted, dehydrated and coverslipped with DPX.

Individual tile images were captured using a Leica M205 FA Stereofluorescence microscope (Leica, Wetzlar, Germany) using a [5× / 0.50 PlanAPO LWD] objective at the equivalent of [64×] magnification and captured using a [DFC 365FX (Leica)] camera through LAS AF v3.1.0.8587software (Leica). Each slide contained 35 tiles to generate a grid which was stitched together using the auto-stitch algorithm.

High definition images were acquired on a Hamamatsu NanoZoomer s360 slide-scanner (Hamamatsu Photonics, Shizuoka, Japan) using a 20× objective. Snapshots of the slide-scans were taken using the Case Viewer software (3D-Histech).

### Quantitative analysis of immunohistochemical staining

Levels of GFP immunohistochemical staining were measured by quantitative threshold image analysis in [Fiji ImageJ (http://imagej.net/Fiji/Downloads)]. Foreground immunostaining was defined by utilising the highest and lowest GFP immunoreactivities across the sections and measured on a scale from 0 (100% transmitted light) to 255 (0% transmitted light) for each pixel. This threshold setting was applied as a constant to all images. Levels of GFP was expressed as mean grey value per brain section.

### GFP Immunofluorescence

For immunofluorescent staining of 50 µm brain sections the following primary antibodies were used: chicken anti-GFP (1:1000, ab13970, Abcam, Cambridge, UK), rabbit anti-NeuN (1:500, ab177487, Abcam, Cambridge, UK), rabbit anti-GFAP (1:1500, Z-0334, Agilent Technologies, Cheshire, UK), and rabbit anti-Iba1 (1:1000, 019-19741, Fujifilm Wako Chemicals, Neuss, Germany). Alexa-conjugated secondary antibodies goat anti-rabbit 488 and goat anti-chicken 594 were used.

Images were collected on a Leica SP8x AOBS Inverted confocal using a 10x / 0.22 HI Plan I Dry objective. The confocal settings were as follows, pinhole 1 airy unit, scan speed 700Hz unidirectional, format 2024 x 2024 pixels. Images were collected using HyD and PMT detectors with the following detection mirror settings: EGFP 493-580nm, AF594 604-699nm; using the white light laser with 488nm (5%) and 594nm (5%) laser lines respectively. When acquiring 3D optical stacks the confocal software was used to determine the optimal number of Z sections. Only the maximum intensity projections of these 3D stacks are shown in the results

### HGSNAT activity assay

For HGSNAT assays, tissue samples were homogenised and sonicated in homogenization buffer (0.5 mol/L NaCl, 0.02 mol/L Tris pH7–7.5), then centrifuged at 2200*g* for 15 minutes at 4 °C, and the supernatant was collected. Protein concentration was determined using Pierce BCA assay kit (Fisher Scientific, Loughborough, UK). HGSNAT activity was measured using 4-methylumbelliferyl-β-D-N-glucosaminide (MU-βGlcNH2, Carbosynth, Compton, UK) as a substrate in duplicates in a black 96-well plate. The assay was performed according to manufacturer’s instructions. Control wells contained 10 μL H_2_O, 10 μL substrate and 10 μL acetyl co-enzyme A whilst sample wells contained 10 μL sample, 10 μL substrate and 10 μL acetyl co-enzyme A. The 96-well plate was covered by a plastic seal and incubated for 18 hours at 37 °C. To terminate the reaction 200 μL carbonate buffer pH 9.5 was added to each well. 4-MU standards (43 μM - 0.17 μM) were added to the plate, and the plate was read for fluorescence at excitation 260 nm and emission 450 nm. Activity was reported as μM 4-MU generated/mg protein/18 hours.

### Vector copy number determination

Analysis of vector biodistribution was performed by quantitative PCR (qPCR). Genomic DNA from tissue homogenates was extracted using the DNeasy blood and tissue kit (Qiagen, Manchester, UK). For quantification of AAV vector copy numbers, a standard curve was prepared by adding specific amounts of linearised AAV-coHGSNAT plasmid and compared against GAPDH using naïve genomic ovine DNA.The primer sequences used were 5’- GGGTCATTAGTTCATAGCCCATA-3’ and 3’-GCCAAGTAGGAAAGTCCCATAA-5’. SYBR Green amplifications were performed using the PowerUp SYBR Green master mix (Fisher Scientific, Loughborough, UK) to a final volume of 25 µL, including 5 µL of DNA. Plasmid amounts were calculated to give the numbers of vector genomes per diploid genomic equivalent.

### IgG antibody assays

Total IgG antibody responses against AAV capsid proteins in serum were measured via ELISA. For each assay, the plates were coated with the same viral vector that the sheep were injected with. The AAV vectors of either serotype was diluted in coating buffer (0.1M NaHCO_3_, pH8.5). The virus was loaded to each well of the 96-well plate and the plates incubated overnight at 4 °C. The plate was washed with wash buffer (PBS, 0.1% Tween-20). Non-specific binding was blocked with blocking buffer (1% BSA 0.02M Tris/HCl, 025M NaCl, pH 7.0) for 1 hour at room temperature. Eight 2-fold serial dilutions were prepared with dilution buffer (PBS, 0.05% Tween, 0.01% BSA) for each serum sample with a starting protein concentration of 10 µg, 50 µL of each serum dilution was applied to the plate in duplicate and incubated for 1 hour at room temperature. After washing, 100 µL of 5 µg/ml biotinylated goat anti-sheep IgG antibody (Vector Laboratories, Cambridge, UK) in dilution buffer was added to each well and incubated at room temperature for 1 hour, aspirated and washed. Each well was incubated with 100 µL of Vectastain ABC kit (Vector Laboratories, Cambridge, UK) for 30 minutes at room temperature and washed. 100 µL of Tetramethylbenzidine substrate was loaded to each well and incubated, 3 minutes at room temperature. The reaction was stopped by adding 50 µL of 2.5 M H_2_SO_4_ to each well. Light absorbance was read at 450 nm to determine the maximum absorbance and at 570 nm to correct for measurement errors on a Synergy HT microplate spectrophotometer (Biotek, Swindon, UK) with Gen5 software.

### Statistical analysis

Statistical analysis was performed using Graphpad Prism software (Graphpad software Inc., San Diego, USA). All data were analysed by analysis of variance (ANOVA) and Tukey post -hoc test for analysis. Significance was assumed at p < 0.05.

## Supplementary Information

**Additional file 1: Fig. S1.** Comparative distribution of GFP in Level 1 in study 1 sheep brains. Images showing GFP expression (brown staining) in all sheep 3 weeks post-injection. Sheep received AAV9-GFP into four locations (10 μL/min), the lateral ventricles (ICV), corpus striatum (CS) and white matter (WM), with the latter two groups separated into two infusion flow rates (0.5 µL.min and 1μL/min).

**Additional file 2: Fig. S2.** Comparative distribution of GFP in Level 2 in study 1 sheep brains. Images showing GFP expression (brown staining) in all sheep 3 weeks post-injection. Sheep received AAV9-GFP into four locations (10 μL/min), the lateral ventricles (ICV), corpus striatum (CS) and white matter (WM), with the latter two groups separated into two infusion flow rates (0.5 µL.min and 1 μL/min).

**Additional file 3: Fig. S3.** Comparative distribution of GFP in Level 3 in study 1 sheep brains. Images showing GFP expression (brown staining) in all sheep 3 weeks post-injection. Sheep received AAV9-GFP into four locations (10 μL/min), the lateral ventricles (ICV), corpus striatum (CS) and white matter (WM), with the latter two groups separated into two infusion flow rates (0.5 µL.min and 1 μL/min).

**Additional file 4: Fig. S4.** Comparative distribution of GFP in Level 4 in study 1 sheep brains. Images showing GFP expression (brown staining) in all sheep 3 weeks post injection. Sheep received AAV9-GFP into four locations (10 μL/min), the lateral ventricles (ICV), corpus striatum (CS) and white matter (WM), with the latter two groups separated into two infusion flow rates (0.5 µL.min and 1 μL/min).

**Additional file 5: Fig. S5.** Comparative distribution of GFP in Level 5 in study 1 sheep brains. Images showing GFP expression (brown staining) in all sheep 3 weeks post-injection. Sheep received AAV9-GFP into four locations (10 μL/min), the lateral ventricles (ICV), corpus striatum (CS) and white matter (WM), with the latter two groups separated into two infusion flow rates (0.5 μL.min and 1 μL/min).

**Additional file 6: Fig. S6.** Comparative distribution of GFP in Level 1 in study 2 sheep brains. Images showing GFP expression (brown staining) in all sheep 3 weeks post injection. Two groups received either AAV9-GFP or AAV-TT-GFP into the corpus striatum (CS) only. An additional group of sheep received AAV-TT-GFP into the CS, white matter (WM) and brainstem (BS).

**Additional file 7: Fig. S7.** Comparative distribution of GFP in Level 2 in study 2 sheep brains. Images showing GFP expression (brown staining) in all sheep 3 weeks post injection. Two groups received either AAV9-GFP or AAV-TT-GFP into the corpus striatum (CS) only. An additional group of sheep received AAV-TT-GFP into the CS, white matter (WM) and brainstem (BS).

**Additional file 8: Fig. S8.** Comparative distribution of GFP in Level 3 in study 2 sheep brains. Images showing GFP expression (brown staining) in all sheep 3 weeks post-injection. Two groups received AAV9-GFP or AAV-TT-GFP into the corpus striatum (CS) only. An additional group of sheep received AAV-TT-GFP into the CS, white matter (WM) and brainstem (BS).

**Additional file 9: Fig. S9.** Comparative distribution of GFP in Level 4 in study 2 sheep brains. Images showing GFP expression (brown staining) in all sheep 3 weeks post-injection. Two groups received AAV9-GFP or AAV-TT-GFP into the corpus striatum (CS) only. An additional group of sheep received AAV-TT-GFP into the CS, white matter (WM) and brainstem (BS).

**Additional file 10: Fig. S10.** Comparative distribution of GFP in Level 5 in study 2 sheep brains. Images showing GFP expression (brown staining) in all sheep 3 weeks post-injection. Two groups received either AAV9-GFP or AAV-TT-GFP into the corpus striatum (CS) only. An additional group of sheep received AAV-TT-GFP into the CS, white matter (WM) and brainstem (BS).

**Additional file 11: Fig. S11.** GFP expression in AAV-GFP treated sheep. There was an absence of GFP expression in GFAP+ astrocytes and Iba1+ microglia/macrophages in AAV9 and AAV-TT treated sheep. Scale bar = 100 µm

**Additional file 12: Fig. S12.** HGSNAT activity in peripheral organs in AAV9-HGSNAT treated sheep. No differences were observed in AAV9-HGSNAT treated sheep *vs* un-injected control sheep 3 weeks after injection.

**Additional file 13: Table S1.** VCN levels in the peripheral organs in AAV9-HGSNAT treated sheep

## Data Availability

All data generated or analysed during this study are included in this published article [and its supplementary information files].

## Abbreviations

AAV: Adeno-associated virus
ANOVA: Analysis of variance
BCA: Bicinchoninic acid
BS: Brainstem
BSA: Bovine serum albumin
CAG: Cytomegalovirus immediate enhancer/β-actin
CED: Convection enhanced delivery
CMV: Cytomegalovirus
CNS: Central nervous system
CS: Corpus striatum
CSF: Cerebrospinal fluid
CT: Computerized tomography
DNA: Deoxyribonucleic acid
ELISA: Enzyme-linked immunosorbent assay
GFAP: Glial fibrillary acidic protein
GFP: Green fluorescent protein
HEK 293: Human embryonic kidney 293 cells
HGSNAT: Heparan sulphate acetyl-CoA: α-glucosaminide N-acetyltransferase
HS: Heparan sulphate
Iba1: Ionized calcium binding adaptor molecule 1
ICV: Intracerebroventricular
IgG: Immunoglobulin G
ITR: Inverted terminal repeats
MPS: Mucopolysaccharidosis
MRI: Magnetic resonance imaging
NeuM: Neuronal nuclear protein
NGS: Normal goat serum
PBS: Phosphate-buffered saline
qPCR: Quantitative PCR
SC: Subcortical zone
SDS PAGE: Sodium dodecyl-sulfate polyacrylamide gel electrophoresis
SMN1: Survival of motor neuron 1
TBS: Tris-buffered saline
VCN: Vector copy number
WM: White matter
