# Supplementary figures and images for: Intraparenchymal convection enhanced delivery of AAV in sheep to treat Mucopolysaccharidosis IIIC
Source: J Transl Med. 2023 Jul 5;21:437. doi: 10.1186/s12967-023-04208-1 (PMC10320977; doi:10.1186/s12967-023-04208-1)

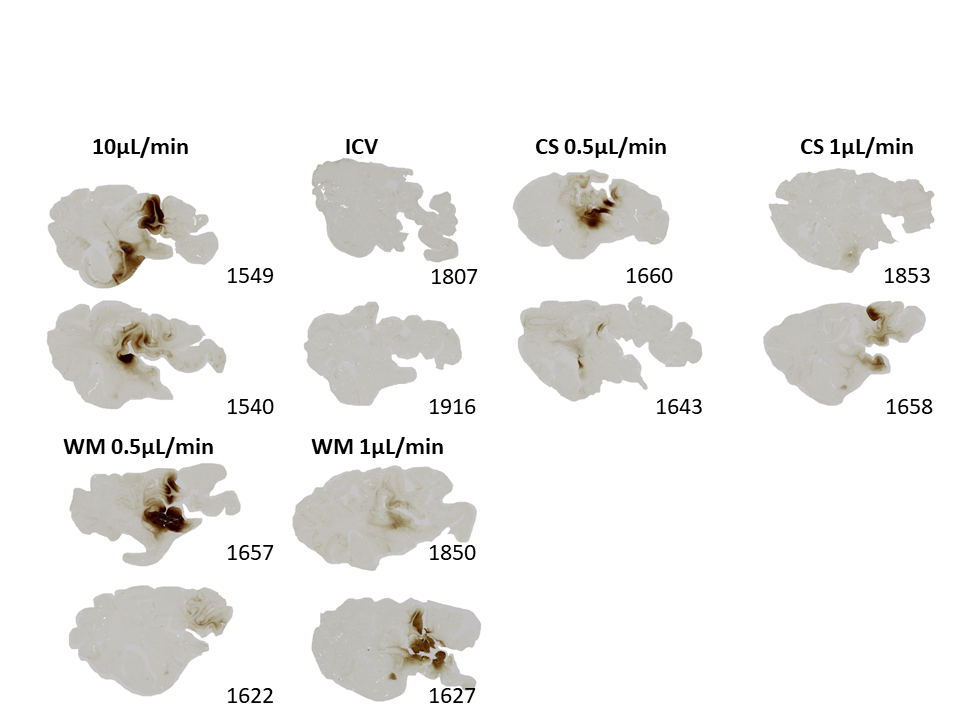

Supplement: Supplementary file 1 — Additional file 1: Fig. S1. Comparative distribution of GFP in Level 1 in study 1 sheep brains. Images showing GFP expression (brown staining) in all sheep 3 weeks post-injection. Sheep received AAV9-GFP into four locations (10 μL/min), the lateral ventricles (ICV), corpus striatum (CS) and white matter (WM), with the latter two groups separated into two infusion flow rates (0.5 µL.min and 1μL/min). [file 12967_2023_4208_MOESM1_ESM.tif]

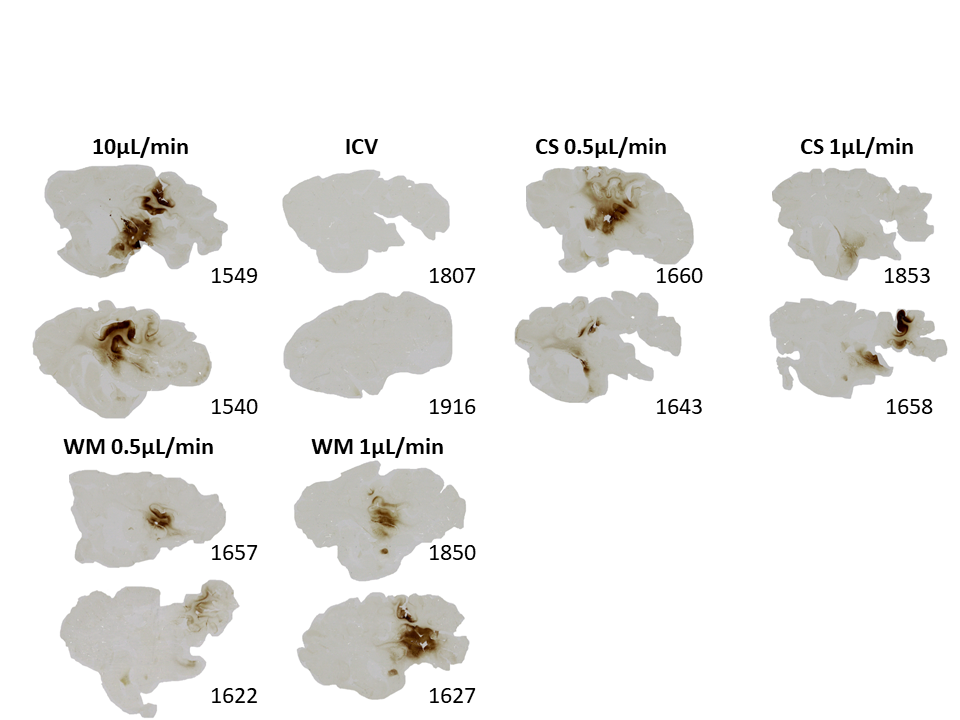

Supplement: Supplementary file 2 — Additional file 2: Fig. S2. Comparative distribution of GFP in Level 2 in study 1 sheep brains. Images showing GFP expression (brown staining) in all sheep 3 weeks post-injection. Sheep received AAV9-GFP into four locations (10 μL/min), the lateral ventricles (ICV), corpus striatum (CS) and white matter (WM), with the latter two groups separated into two infusion flow rates (0.5 µL.min and 1 μL/min). [file 12967_2023_4208_MOESM2_ESM.tif]

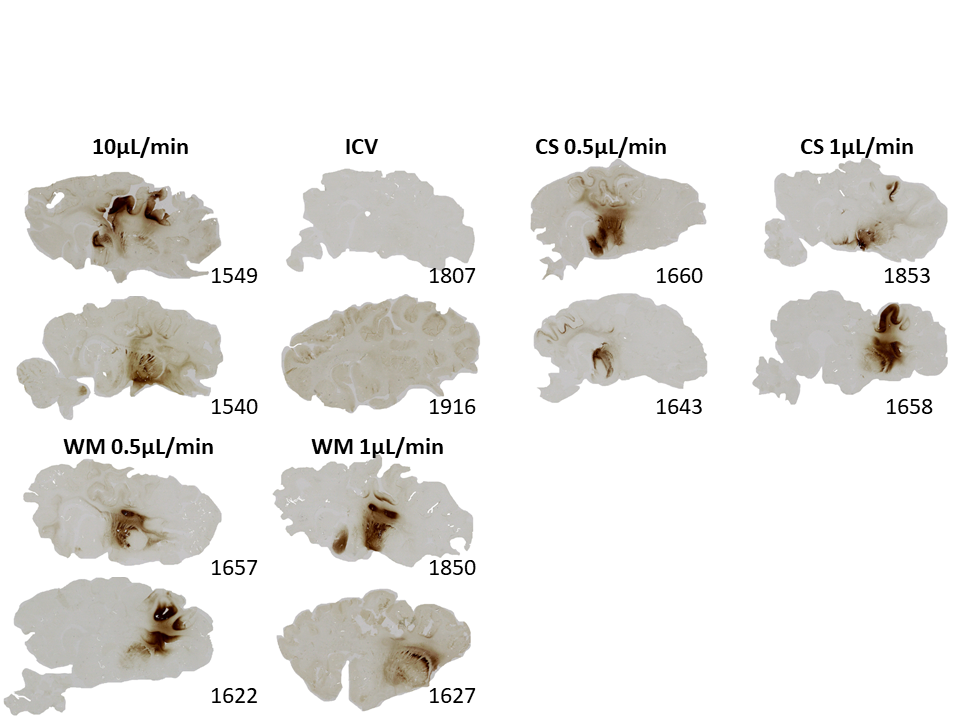

Supplement: Supplementary file 3 — Additional file 3: Fig. S3. Comparative distribution of GFP in Level 3 in study 1 sheep brains. Images showing GFP expression (brown staining) in all sheep 3 weeks post-injection. Sheep received AAV9-GFP into four locations (10 μL/min), the lateral ventricles (ICV), corpus striatum (CS) and white matter (WM), with the latter two groups separated into two infusion flow rates (0.5 µL.min and 1 μL/min). [file 12967_2023_4208_MOESM3_ESM.tif]

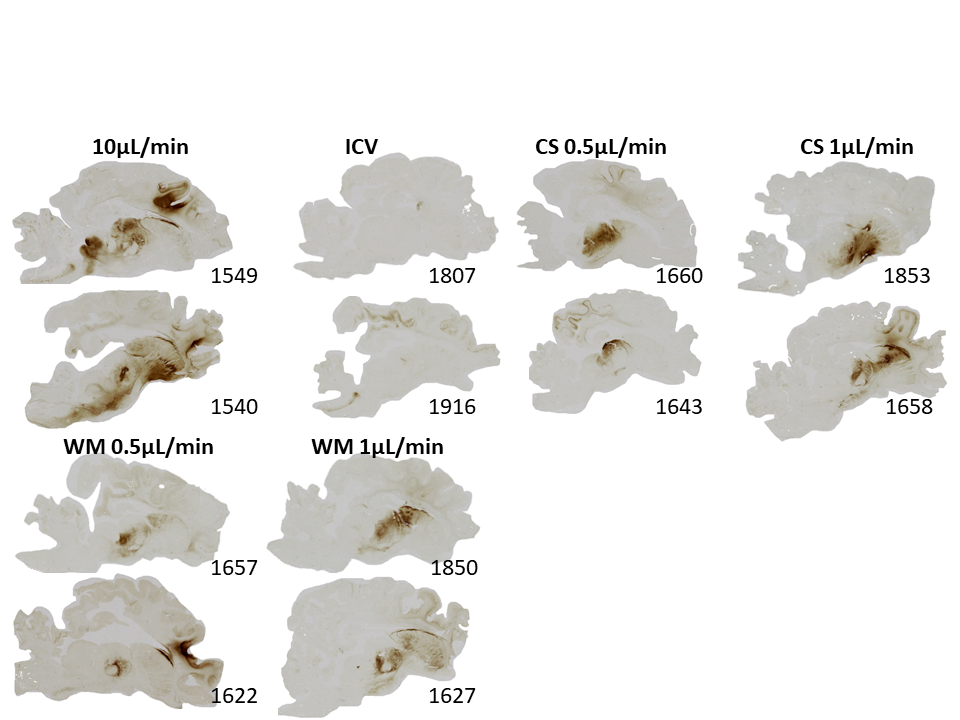

Supplement: Supplementary file 4 — Additional file 4: Fig. S4. Comparative distribution of GFP in Level 4 in study 1 sheep brains. Images showing GFP expression (brown staining) in all sheep 3 weeks post injection. Sheep received AAV9-GFP into four locations (10 μL/min), the lateral ventricles (ICV), corpus striatum (CS) and white matter (WM), with the latter two groups separated into two infusion flow rates (0.5 µL.min and 1 μL/min). [file 12967_2023_4208_MOESM4_ESM.tif]

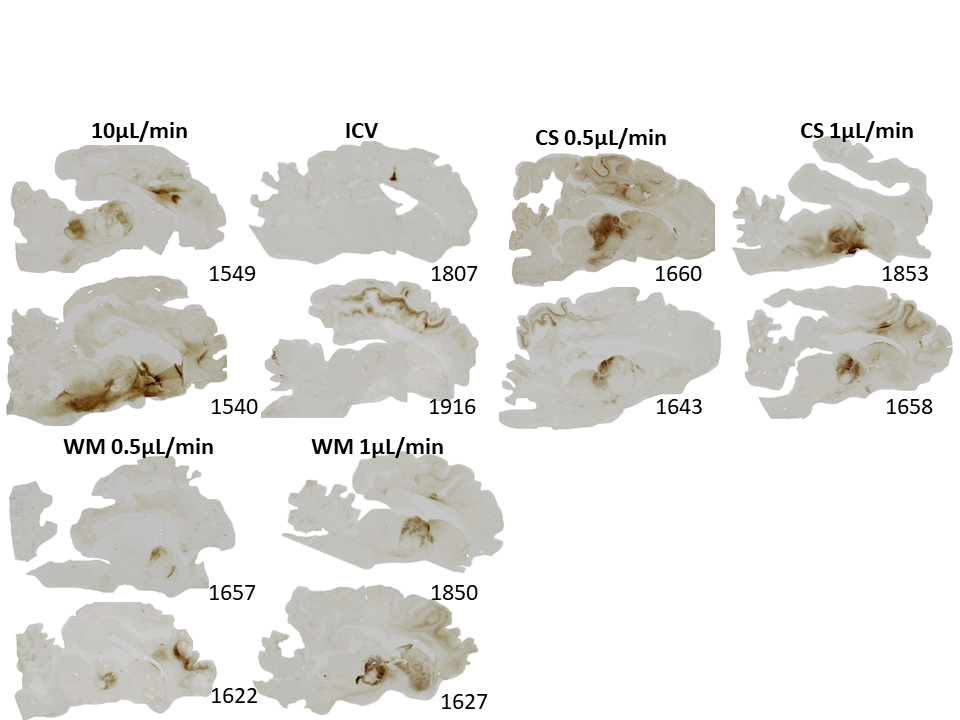

Supplement: Supplementary file 5 — Additional file 5: Fig. S5. Comparative distribution of GFP in Level 5 in study 1 sheep brains. Images showing GFP expression (brown staining) in all sheep 3 weeks post-injection. Sheep received AAV9-GFP into four locations (10 μL/min), the lateral ventricles (ICV), corpus striatum (CS) and white matter (WM), with the latter two groups separated into two infusion flow rates (0.5 μL.min and 1 μL/min). [file 12967_2023_4208_MOESM5_ESM.tif]

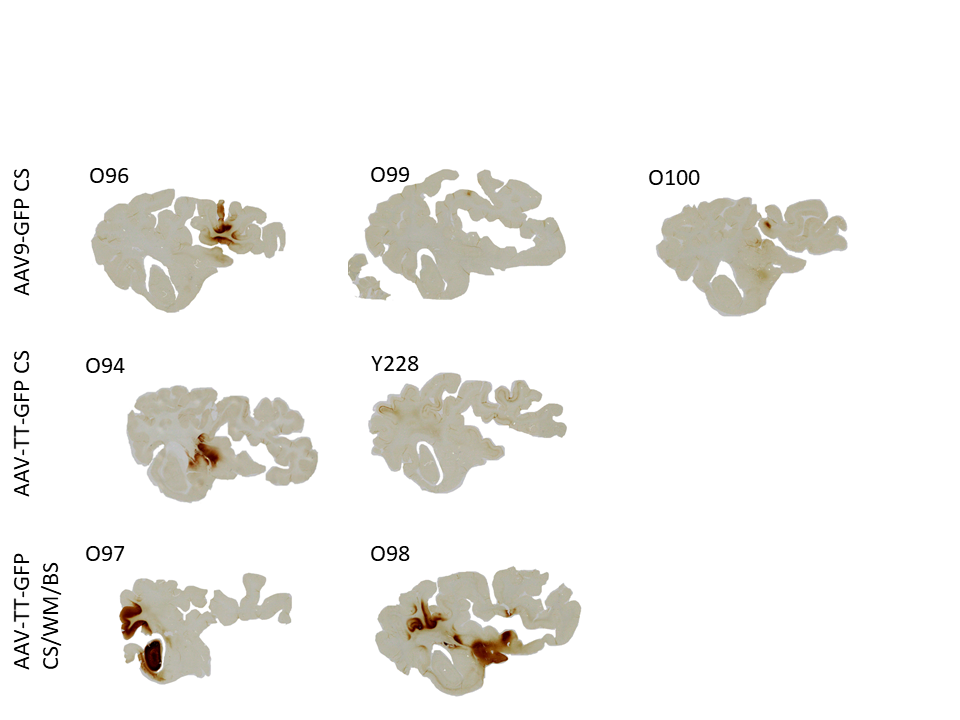

Supplement: Supplementary file 6 — Additional file 6: Fig. S6. Comparative distribution of GFP in Level 1 in study 2 sheep brains. Images showing GFP expression (brown staining) in all sheep 3 weeks post injection. Two groups received either AAV9-GFP or AAV-TT-GFP into the corpus striatum (CS) only. An additional group of sheep received AAV-TT-GFP into the CS, white matter (WM) and brainstem (BS). [file 12967_2023_4208_MOESM6_ESM.tif]

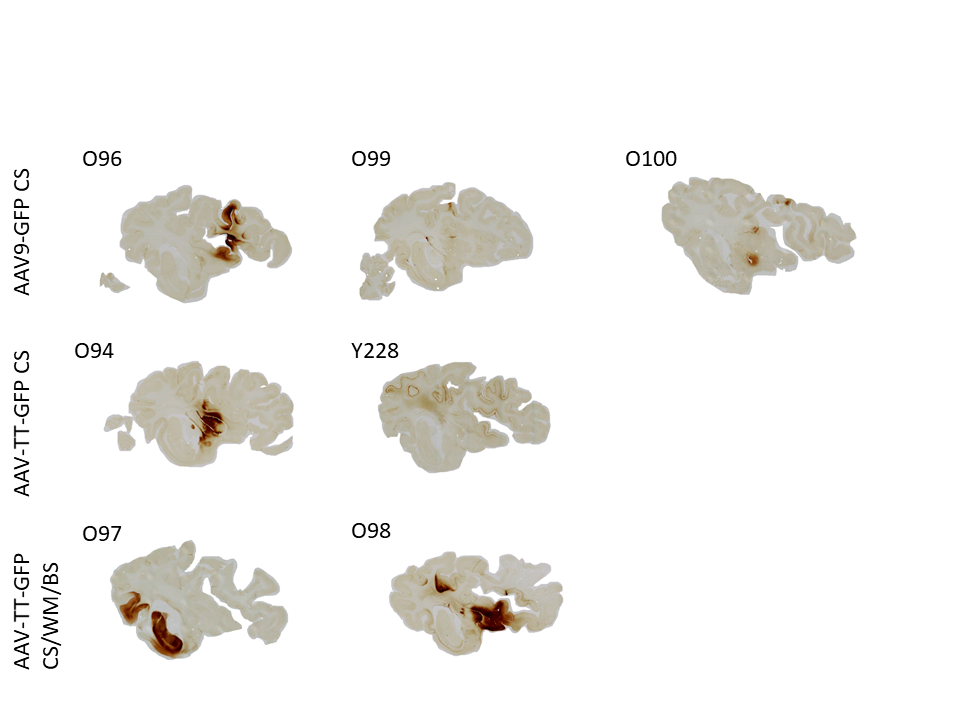

Supplement: Supplementary file 7 — Additional file 7: Fig. S7. Comparative distribution of GFP in Level 2 in study 2 sheep brains. Images showing GFP expression (brown staining) in all sheep 3 weeks post injection. Two groups received either AAV9-GFP or AAV-TT-GFP into the corpus striatum (CS) only. An additional group of sheep received AAV-TT-GFP into the CS, white matter (WM) and brainstem (BS). [file 12967_2023_4208_MOESM7_ESM.tif]

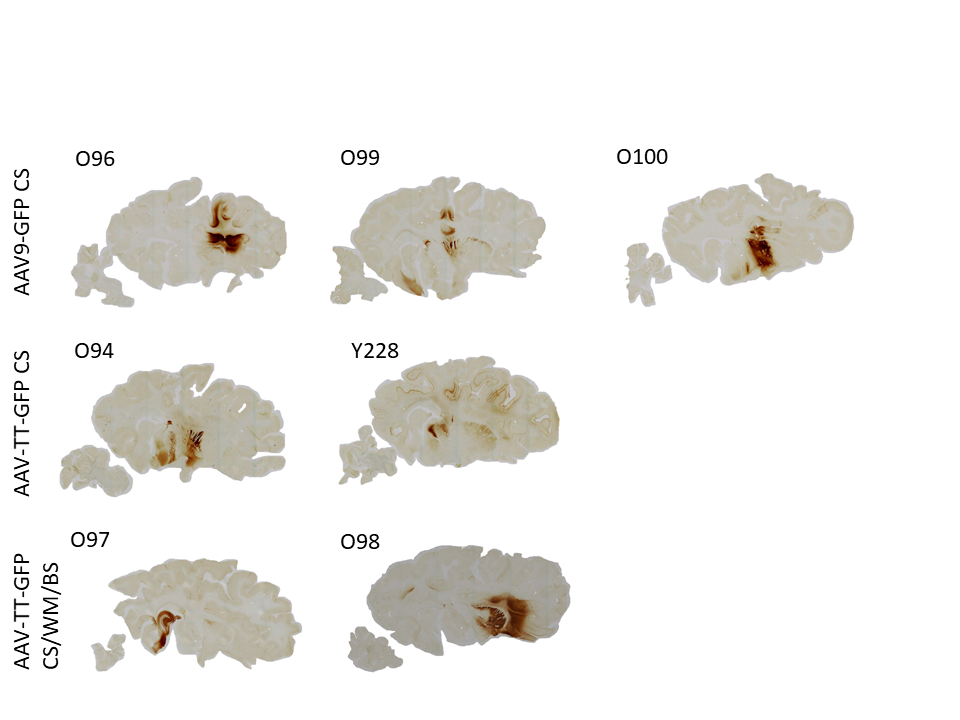

Supplement: Supplementary file 8 — Additional file 8: Fig. S8. Comparative distribution of GFP in Level 3 in study 2 sheep brains. Images showing GFP expression (brown staining) in all sheep 3 weeks post-injection. Two groups received AAV9-GFP or AAV-TT-GFP into the corpus striatum (CS) only. An additional group of sheep received AAV-TT-GFP into the CS, white matter (WM) and brainstem (BS). [file 12967_2023_4208_MOESM8_ESM.tif]

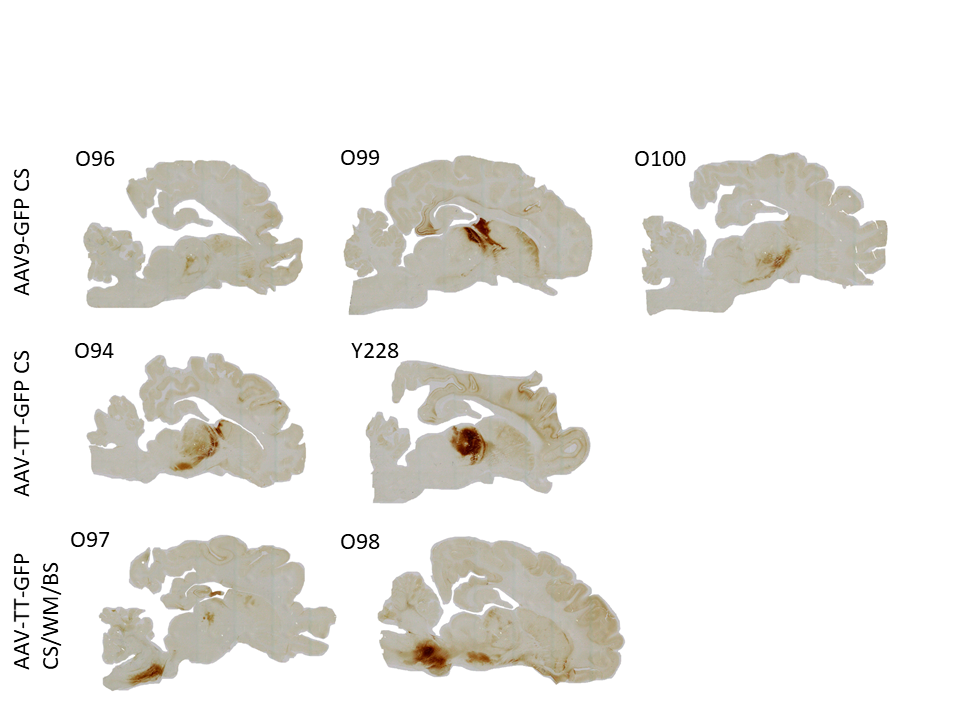

Supplement: Supplementary file 9 — Additional file 9: Fig. S9. Comparative distribution of GFP in Level 4 in study 2 sheep brains. Images showing GFP expression (brown staining) in all sheep 3 weeks post-injection. Two groups received AAV9-GFP or AAV-TT-GFP into the corpus striatum (CS) only. An additional group of sheep received AAV-TT-GFP into the CS, white matter (WM) and brainstem (BS). [file 12967_2023_4208_MOESM9_ESM.tif]

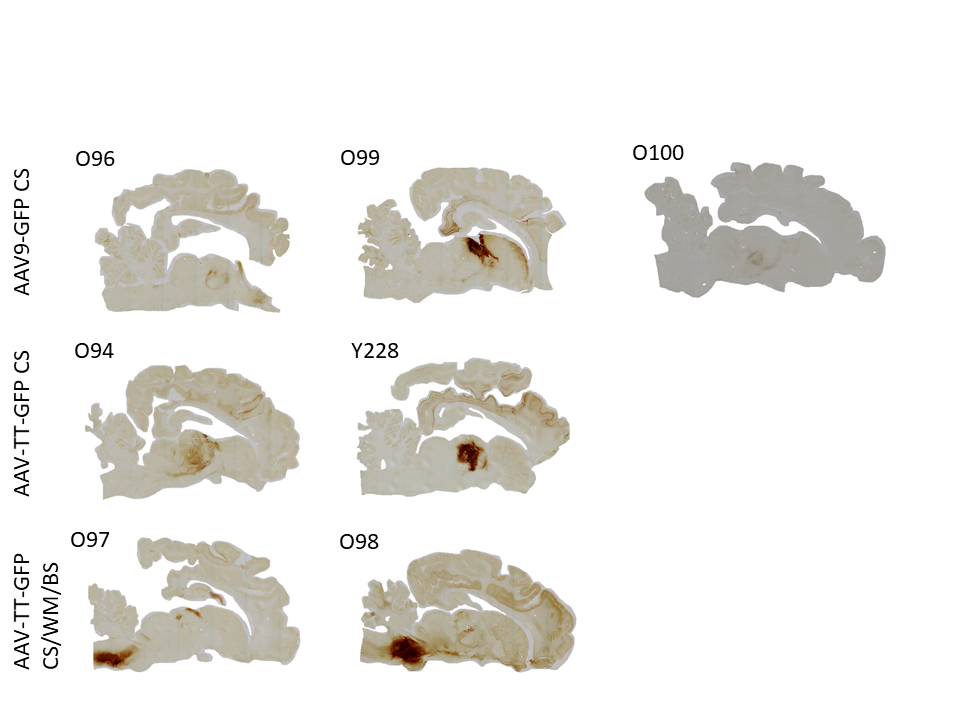

Supplement: Supplementary file 10 — Additional file 10: Fig. S10. Comparative distribution of GFP in Level 5 in study 2 sheep brains. Images showing GFP expression (brown staining) in all sheep 3 weeks post-injection. Two groups received either AAV9-GFP or AAV-TT-GFP into the corpus striatum (CS) only. An additional group of sheep received AAV-TT-GFP into the CS, white matter (WM) and brainstem (BS). [file 12967_2023_4208_MOESM10_ESM.tif]
