# Supplementary material for: Intraparenchymal convection enhanced delivery of AAV in sheep to treat Mucopolysaccharidosis IIIC
Source: J Transl Med. 2023 Jul 5;21:437. doi: 10.1186/s12967-023-04208-1 (PMC10320977; doi:10.1186/s12967-023-04208-1)
Supplement: Supplementary file 13 — Additional file 13: Table S1. VCN levels in the peripheral organs in AAV9-HGSNAT treated sheep [file 12967_2023_4208_MOESM13_ESM.docx]

| **Organ** | **O95 (vg/cell)** | **Y242 (vg/cell)** |
| --- | --- | --- |
| Bladder | 0.00 | 0.00 |
| Inflamed mesenteric lymph node | 0.00 | 0.00 |
| Kidney | 0.01 | 0.00 |
| Liver | 0.00 | 0.00 |
| Lung | 0.00 | 0.00 |
| Lymph node | 0.00 | 0.00 |
| Spleen | 0.00 | 0.00 |
| Bone marrow | 0.00 | 0.01 |
| Ovary | 0.00 | 0.01 |
| Heart | 0.04 | 0.10 |
| Oesophagus | 0.00 | 0.07 |
| Spinal cord (cervical) | 0.03 | 0.04 |
| Spinal cord (lumbar) | 0.00 | 0.01 |
| Spinal cord (thoracic) | 0.00 | 0.01 |
| Small intestine | 0.00 | 0.00 |
| Trachea | 0.00 | 0.00 |
| Pancreas | 0.00 | 0.25 |

**Table S1. VCN levels in the peripheral organs in AAV9-HGSNAT treated sheep.**
